# Room-Temperature One-Pot Synthesis of pH-Responsive Pyridine-Functionalized Carbon Surfaces

**DOI:** 10.1021/acsomega.2c06847

**Published:** 2023-03-17

**Authors:** Isobel M. Wilson, Sandeep K. Padamati, Antonia D. Bobitan, Michael J. Porter, Katherine B. Holt

**Affiliations:** Department of Chemistry, University College London, 20 Gordon St., London WC1H 0AJ, U.K.

## Abstract

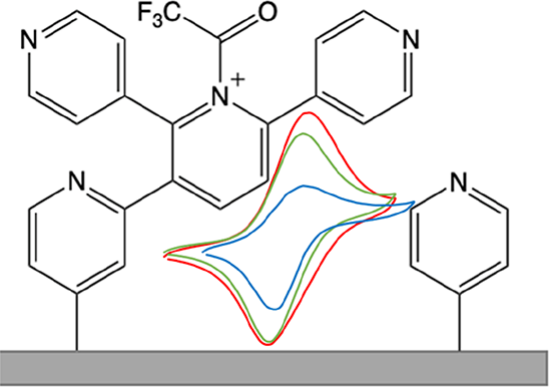

Carbon surfaces (glassy carbon, graphite, and boron-doped diamond) were functionalized with layers composed of linked pyridinium and pyridine moieties using simple electrochemical reduction of trifluoroacetylpyridinium. The pyridinium species was generated *in situ* in solution by the reaction of trifluoroacetic anhydride and pyridine precursors and underwent electrochemical reduction at −1.97 V vs Fc/Fc^+^, as determined by cyclic voltammetry. The pyridine/pyridinium films were electrodeposited at room temperature, on a timescale of minutes, and were characterized using X-ray photoelectron spectroscopy. The as-prepared films have a net positive charge in aqueous solution at pH 9 and below due to the pyridinium content, confirmed by the electrochemical response of differently charged redox molecules at the functionalized surfaces. The positive charge can be enhanced further through protonation of the neutral pyridine component by controlling the solution pH. Moreover, the nitrogen–acetyl bond can be cleaved through base treatment to purposefully increase the neutral pyridine proportion of the film. This results in a surface that can be “switched” from functionally near neutral to a positive charge by treatment in basic and acidic solutions, respectively, through manipulation of the protonation state of the pyridine. The functionalization process demonstrated here is readily achievable at a fast timescale at room temperature and hence can allow for rapid screening of surface properties. Such functionalized surfaces present a means to test in isolation the specific catalytic performance of pyridinic groups toward key processes such as oxygen and CO_2_ reduction.

## 1 Introduction

Nitrogen-doped carbon materials have attracted recent attention as potential metal-free electrocatalysts for the oxygen reduction^1^ and CO_2_ reduction^2^ reactions, and for lithium-ion battery anodes^3^ and electrochemical glucose sensing,^4^ among other applications.^5^ Such nitrogen-doped materials include graphene,^6^ carbon nanotubes,^7^ activated carbon,^8^ aerogels,^9^ pyrolyzed photoresist film (PPF),^10^ and carbon black.^11^ Nitrogen can be incorporated into the carbon structure substitutionally (graphitic nitrogen) or as pyrrolic and pyridinic functionalities. While some control over the relative proportions of the nitrogen functionalities can be achieved through precursor selection and reaction conditions, it is difficult to synthesize materials with only one type of nitrogen bonding environment. Achieving this would enable the relative activities of the different nitrogen environments toward key catalytic processes such as oxygen reduction to be explored in isolation,^12^ enabling rational design of more effective catalyst materials with optimized concentrations and locations of active sites.

Most methods for introducing nitrogen functionalities into or onto carbon require high temperatures, for example, chemical vapor deposition,^13^ carbonization of N-containing carbon-based polymers,^14^ pyrolysis of carbon- and nitrogen-containing precursors,^15^ and arc-discharge from graphite electrodes.^16^ As stated above, these methods are generally not successful in selecting for one nitrogen bonding environment over another. A recent exception is N-doped hydrogen-substituted graphdiyne,^17^ where the nitrogen species are solely located on graphitic edge-type sites. This was successfully synthesized and used to study the specific catalytic activity of pyridinic nitrogen toward the oxygen reduction reaction. However, the synthesis of this material is complex, so it is desirable to explore other more facile methods for the selective modification of carbon with heteroatom functionalities so that their reactivity can be investigated more systematically.

Other recent studies have proposed that decoration of carbon surfaces with pyridine through a covalent linkage, rather than bulk doping of the material, is a better way to achieve a homogeneous nitrogen environment for catalysis testing, as demonstrated for carbon nanotubes.^18^ The rationale for this approach is that catalysis is mediated through surface sites, so modification of the surface, rather than bulk doping, is a more effective means to achieve high surface coverage of active sites. In this example, diazonium salts were used to modify carbon nanotubes with different pyridine moieties, to allow a systematic study of the activity of the functionalities toward oxygen reduction as a function of the electronic structure.^19^ Functionalization of carbon nanotubes has been demonstrated through a reaction with diazopyridinium cations, generated *in situ* through a reaction between aminopyridine compounds and sodium nitrite in acidic solution, although lengthy reaction times (19 h) at low temperatures (0–5 °C) are necessary.^20^ Alternatively, electrochemical reduction of *in situ* generated diazonium species is a well-established route to rapidly modify carbon electrodes with pyridine.^21,22^ Subsequent electrochemical reduction of diazopyridinium cations results in multilayer electrochemical grafting of pyridine to carbon^23^ and has also been demonstrated for other substrates such as platinum,^24^ gold,^21^ and silicon.^25^ However, diazopyridinium cations have limited stability in water, undergoing a reaction to hydroxypyridine within minutes at room temperature; indeed, within 1 h, 95% of 3-diazopyridinium cations were found to be converted to 3-hydroxypyridine.^21^ Hence, electrochemical grafting of pyridine using *in situ* generated diazopyridinium cations must be undertaken within 2 min of mixing of precursors if carried out at room temperature,^21,23^ or alternatively, low temperatures (0–5 °C) must be used.^22,26^

**Scheme 1:**
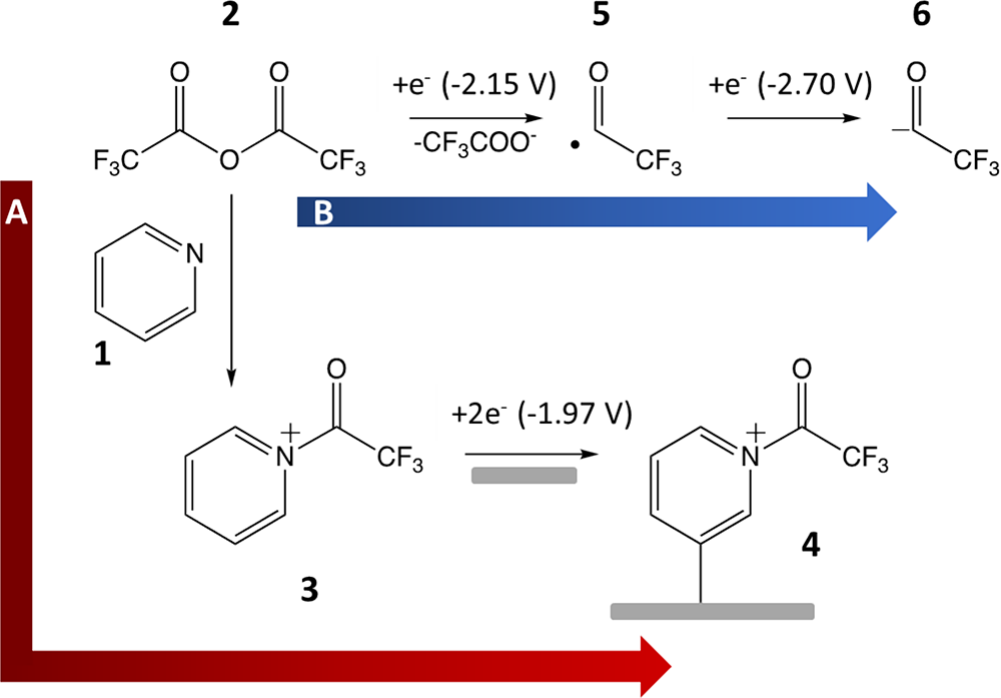
Proposed Reaction Scheme for the Reaction of Pyridine (**1**) with TFAA (**2**) to Form a Proposed Pyridinium Salt (**3**), Which Was Then Electrochemically Reduced Resulting in Proposed Surface-Bound Species (**4**) via Route A; Route B Shows the Reduction of **2** in the Absence of Pyridine to Give a Trifluoroacetyl Radical (**5**) and an Acyl Anion (**6**); Potentials Are vs Fc/Fc^+^

In this paper, we describe a new rapid one-pot room-temperature approach for the modification of different carbon electrode materials (glassy carbon, graphite, and boron-doped diamond) by electrochemical reduction of *in situ* generated pyridinium salts (see Scheme 1). We reacted pyridine (**1**) with trifluoroacetic anhydride (TFAA, **2**) to form a proposed trifluoroacetylpyridinium salt (**3**) that can undergo electrochemical reduction and graft to a carbon electrode to form a surface species (**4**). This method is highly effective because it generates *in situ* a pyridinium species that can undergo electrochemical reduction at potentials well positive of the solvent reduction window. Pyridine itself cannot be reduced in this potential range, so the formation of the salt is essential as a precursor to the electrochemical reduction. The method is also potentially applicable to other organic heteroatomic species that can form *in situ* salts on addition of TFAA. In contrast to previous modification studies, our approach avoids the utilization of diazonium salts, with the advantages that **3** is stable at room temperature for at least several hours, avoiding the need for the low temperatures or short functionalization timescales required when using diazonium salts.

As described herein, we can produce multilayer films of pyridine electrochemically grafted to the surface. As well as the potential use of such functionalized surfaces for rapid catalytic screening for the oxygen reduction and CO_2_ reduction reactions (among others), pyridine surface modification has previously found application for CO_2_ capture^27^ and the absorption and sensing of toxic metal ions in solution.^28^ Pyridine is also a versatile ligand, lending such surfaces to further modification with metal-centered complexes and other materials.^26^ However, to optimize the application of pyridine-functionalized surfaces in aqueous environments, it is also essential to understand their protonation chemistry; hence, this is a major focus of this paper, as a part of a full characterization of the film properties. Preliminary results showing the potential of these functionalized surfaces for electrochemical CO_2_ reduction are also presented.

## 2 Experimental Methods

### 2.1 Carbon Electrode Materials

Carbon electrode materials used for modification were a 3 mm-diameter glassy carbon (GC) disk mounted in PEEK (BASi), graphite rods of varying length and diameter (Goodfellow), and a boron-doped diamond (BDD) disk of 3 mm diameter sealed in PEEK (Windsor Scientific).

### 2.2 Electrode Modification

Carbon electrodes were modified by electrochemical reduction in *ca.* 10 mM solutions of trifluoroacetylpyridinium (**3**). To make these solutions, 0.1 M TBAPF_6_ in anhydrous acetonitrile (MeCN) was used as the electrolyte solution for the following electrochemical reduction process. Under constant stirring, pyridine (**1**, 10 mM) was added to the MeCN electrolyte followed by the slow addition of excess TFAA (**2**, 15 mM). The solution was then deoxygenated by bubbling argon gas through for at least 20 min. Before modification, GC and BDD electrodes were polished with a 0.3 μm alumina solution on a Buehler Microcloth polishing pad and then rinsed. Graphite rods were not polished but treated as received. All electrochemical measurements used an Autolab potentiostat (EcoChemie, Netherlands), controlled by GPES version 4.7. The cell was set up using a nickel counter electrode and a silver wire quasireference electrode. The working electrode was the electrode chosen to be modified (GC, graphite, or BDD). Modification of platinum and gold electrodes was attempted, but this was unsuccessful. The electrodes were modified either via a constant potential of −2 V (vs Fc/Fc^+^) or via cycling the potential of the working electrode from −0.5 to −2.5 V (vs Fc/Fc^+^) (number of cycles indicated in the text). All cyclic voltammograms were performed at a scan rate of 0.1 V s^–1^. Before all subsequent electrochemical studies, the electrode was then sonicated in MeCN to remove unbound material, before being left to air-dry.

### 2.3 X-ray Photoelectron Spectroscopy (XPS) Measurements

Graphite rods were modified using the techniques described above, by either cycling for 9 cycles or holding at −2 V, and then sonicated in MeCN for 5 min to remove any unbound material. In some cases, modified rods were sonicated in different solvents (MeCN, acetone, water, and dichloromethane) for up to one hour to determine the physical stability of the films. The graphite rods were then cut to size to fit in the XPS chamber. The modified electrodes were prepared the day before and kept under vacuum overnight before being transferred to the XPS chamber. XPS measurements were taken using a Thermo Scientific K-Alpha instrument with a monochromated microfocused Al Kα X-ray source (1486.6 eV). These measurements were performed under ultrahigh vacuum conditions with a 400 μm spot size. The spectral data acquired from the XPS experiment were then peak fitted using CASA XPS and Origin to identify which species were present on the surface of the graphite electrodes and in what quantities.

### 2.4 Redox Probe Experiments

Potassium hexacyanoferrate(II) (K_4_Fe(CN)_6_, ferrocyanide), hexaamineruthenium(III) chloride (Ru(NH_3_)_6_Cl_3_), and ferrocenemethanol were used as redox probes to investigate the properties of the modified electrodes. All were obtained from Merck and used as received. Solutions (1 mM) of each redox probe were made up with a NaCl (0.1 M) electrolyte in deionized water, and CVs were recorded. The cell was set up using a nickel counter electrode and a silver/silver chloride (Ag/AgCl) reference electrode. The working electrode was the pyridine-functionalized carbon electrode.

### 2.5 pH Dependence

The behavior of the ferrocyanide redox probe at the electrode modified from trifluoroacetylpyridinium was then investigated at a range of different pH values, to provide information on how the charge on the modified electrode surface changed in response to pH. To do this, solutions of 1 mM ferrocyanide were prepared with different 0.1 M phosphate-buffered solutions (PBS, made from different proportions of K_2_HPO_4_ and KH_2_PO_4_) to create solutions of ferrocyanide at a range of pH values. Electrodes that had been modified from trifluoroacetylpyridinium, via a constant potential of −2 V vs Fc/Fc^+^ for 30 s, were then used to conduct cyclic voltammetry in the ferrocyanide solutions. A 30 s modification was chosen as the time required to grow films with maximum enhanced redox response to ferrocyanide (see the SI). The modified electrodes were placed in the ferrocyanide solutions for 5 min before the cyclic voltammograms were taken, to allow the films to be equilibrated to the pH of the solution.

### 2.6 Strong Base/Acid Treatment

The charge on the surface of the modified electrodes was controlled by treating the modified electrodes with a strong base and acid. Two forms of pretreatment were carried out: base treatment (placed in a solution of 1 M NaOH for 3 min) or base/acid treatment (placed in a solution of 1 M NaOH for 3 min, rinsed, and then placed in a solution of 1 M H_2_SO_4_ for 3 min).

### 2.7 Electrochemical CO_2_ Reduction

Unmodified and pyridine-functionalized GC, graphite, and BDD electrodes were used for electrochemical CO_2_ reduction, and the solution phase products were determined using ^1^H NMR (400 MHz, Bruker). Phosphate-buffered solution of pH 7.4 was saturated with CO_2_, and a potential of −1.2 V vs Ag/AgCl was applied. Aliquots of solution were analyzed by NMR at different time intervals. Control experiments were carried out with argon-saturated solution at the same potentials.

### 2.8 Computational Methods

All DFT calculations were carried out using the Gaussian-16 software package.^29^ Geometry optimizations were carried out using the (U)B3LYP functional and the 6-31+G(d,p) basis set, and vibrational frequency calculations were performed on the minimized structures to confirm that they lay on local minima. Solvation effects were accounted for using the CPCM method, with acetonitrile as the solvent, in all calculations.

Redox potentials were calculated as described by Roth *et al*.^30^ according to the equation
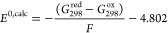
where *E*^0, calc^ is the calculated redox potential, *G*_298_^red/ox^ are the free energies of the reduced and oxidized forms calculated at 298 K, and *F* is Faraday’s constant. The −4.802 V correction term is derived from the absolute value of the standard hydrogen electrode (4.281 V),^31^ the potentials of the saturated calomel electrode (SCE) vs SHE (−0.141 V),^24^ and the ferrocene/ferrocenium couple vs SCE (−0.380 V).^32^ Spin populations were calculated using Multiwfn version 3.8,^33^ by integrating the spin density in fuzzy atomic spaces as defined by Becke.^34^

## 3 Results and Discussion

### 3.1 Functionalization of Carbon with Pyridine by Electrochemical Reduction

**Figure 1:**
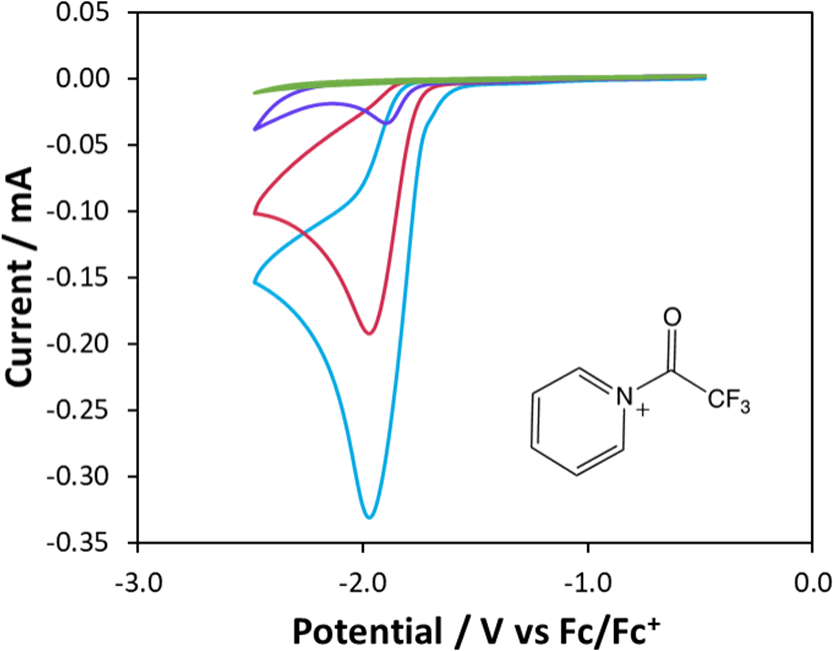
CVs of the proposed trifluoroacetyl pyridium salt (**3**, ca. 10 mM) formed in situ by addition of TFAA (**2**) to pyridine (**1**) in degassed, anhydrous acetonitrile with a 0.1 M TBAPF_6_ electrolyte, a scan rate of 0.1 V s^–1^, and a working electrode 3 mm-diameter GC disk. Scan 1 = light blue; scan 2 = red; scan 3 = purple; scan 9 = green.

As described above, we propose that addition of pyridine (**1**) to TFAA (**2**) results in the formation of a pyridinium salt (**3**); although isolation and *ex situ* characterization of the salt proved difficult, NMR analysis of the solution suggested salt formation (see the SI). Evidence for the salt formation additionally comes from comparison of the CV response for pyridine alone (SI), TFAA alone (SI), and the mixture (Figure 1). Pyridine itself exhibits no CV redox peaks in this potential range, showing that it cannot be reduced under these conditions. TFAA can be reduced, but the CV response is different from that of the mixture. The TFAA response is described in more detail in the SI, but briefly, an initial reduction is observed at −2.15 V where we propose that TFAA is reduced to produce a trifluoroacetyl radical (**5**, see Scheme 1 route B) and a carboxylate. A second reduction step at −2.70 V is attributed to reduction of the radical (**5**) to the acyl anion (**6**).

For the mixture, a single reduction peak is observed in Figure 1 at −1.97 V, indicating that a new species is present in solution, proposed to be the pyridinium salt species (**3**) shown in the inset. When the potential was cycled between −0.5 and −2.5 V, in each consecutive cycle, the peak reduction current decreases, indicating that the electrode surface is becoming passivated. Beyond scan 4, the reduction peak was barely observed, indicating the presence of a film on the electrode surface, which is inhibiting the electron transfer. We propose that **3** undergoes reduction to form a radical species that can graft to the carbon electrode surface to form a species similar to **4**. We propose through analogy with the literature on pyridine modification using diazonium precursors that grafting occurs through formation of a C–C bond (Scheme 1) although further experimental characterization would be required to confirm this. Trapping experiments, using styrene as a radical trap, provide some evidence for radical formation being important in the mechanism (see the SI). Analysis of the composition of the film (discussed below) indicates that further reduction of some of the grafted cationic trifluoroacetylpyridine (**4**) is possible, resulting in cleavage of the N–COCF_3_ bond to leave neutral pyridine functionalities on the surface. Pyridinium radicals formed by subsequent reduction can also react with surface-bound pyridinium or pyridine species to form multilayers, eventually inhibiting further electron transfer. The thickness of the resulting film was estimated by determining the charge passed during nine consecutive CVs recorded between −0.5 and −2.5 V. We estimated that for every 2 electrons passed from the electrode, one molecule of **3** undergoes reduction and then binds directly to the electrode surface or couples with existing bound pyridinium (or pyridine) to form multilayers. The total number of electrons passed during nine CVs was thus calculated to be equivalent to approximately three layers of surface-bound pyridine. This is likely to be an overestimation of thickness as not every molecule that is reduced will undergo grafting, so the most accurate description would be that the film is no thicker than three layers of pyridine. A detailed description of this calculation can be seen in the SI.

The CV of **3** was recorded using other electrode materials, where the response using graphite rods and BDD was similar to using GC, specifically with respect to the rapid decrease in current with consecutive scans, indicating that a layer is being formed at the electrode. In contrast, such passivation was not observed using gold or platinum electrodes, and consecutive CVs were identical to the first (see the SI). Thus, this illustrates that only carbon surfaces undergo functionalization using this method. This suggests that carbon–carbon bond formation with the surface is important in film formation, rather than a simple physical deposition and passivation with polymerized material. This is different from surface modification using diazonium species, where noncarbon substrates can undergo functionalization.^21,24,25^ This mechanistic difference may be due to the delocalized nature of the radical species formed on reduction of **3** (see below) and the requirement for an additional H-abstraction step during the grafting mechanism. In contrast, the radical formed after reduction of the diazonium species is localized on the ring position where N_2_ has been eliminated and is likely to be more reactive and hence less selective in the reaction with a substrate. However, as functionalization of carbon was the focus of this study, we have not considered the grafting reactions at other substrates further. We are also able to demonstrate the modification of carbon surfaces using different substituted pyridine precursor species (see the SI) illustrating the potential versatility of this functionalization approach.

**Figure 2:**
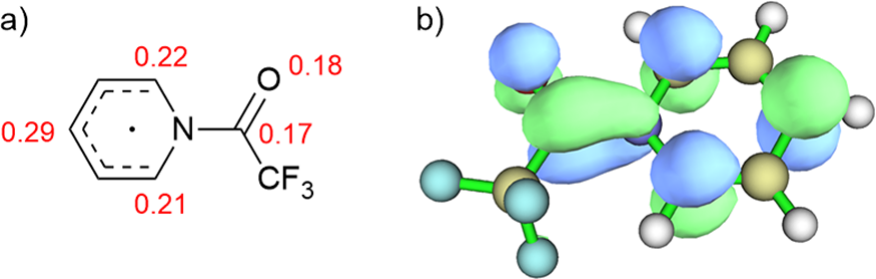
(a) Spin populations in the trifluoroacetylpyridyl radical (calculated with UB3LYP/6-31+G(d,p)); (b) depiction of the SOMO of the trifluoroacetylpyridyl radical.

To validate our supposition that the trifluoroacetylpyridinium ion would be more readily reduced than pyridine or TFAA, we used DFT calculations to predict their redox potentials. The method used was that of Haziri *et al*.,^24^ which has been shown to give good agreement with experimental values for a range of organic compounds, although it should be noted that in their paper, the calculated value for reduction of TFAA showed poor agreement, being more negative than the calculated value by *ca.* 0.5 V. Structures of the reduced and oxidized forms of each species were optimized in MeCN solution, and their free energies were used to calculate redox potentials vs Fc/Fc^+^. The values obtained were TFAA −1.31 V, pyridine −3.15 V, and trifluoroacetylpyridinium −0.19 V, supporting our hypothesis that the trifluoroacetylpyridinium ion would be markedly easier to reduce than either pyridine or TFAA. Calculation of spin populations for the reduced trifluoroacetylpyridyl radical (Figure 2a) indicated that the highest spin populations were at the 2, 4, and 6 positions of the pyridine ring, suggesting that these would be likely sites of the reaction with the carbon surface. Inspection of the SOMO of this radical (Figure 2b) also supported this conclusion. This is a different, more delocalized distribution of electron density than noted for diazonium species, where the highest spin population (and hence the reaction site) is at the position of diazo substitution and may explain the varying reactivity toward different substrates that is observed.

### 3.2 XPS Characterization of Functionalized Carbon Surfaces

**Figure 3:**
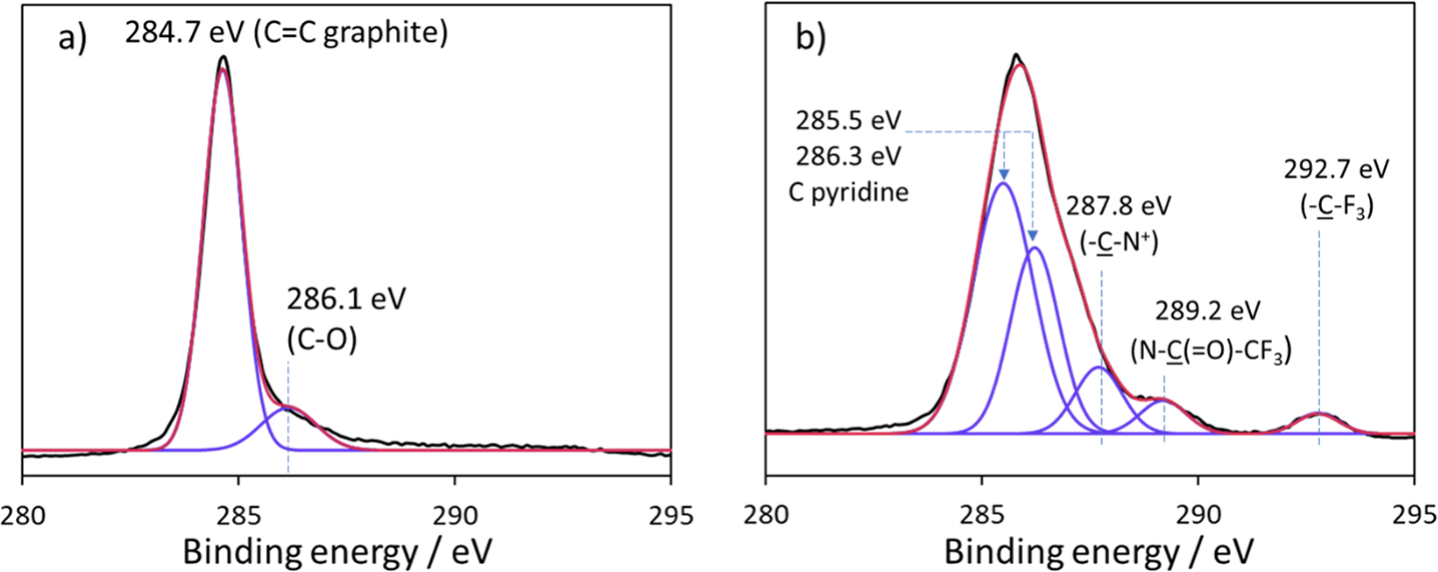
C 1s region of (a) unmodified graphite and (b) graphite modified with trifluoroacetylpyridinium after electrochemical cycling followed by 1 h of sonication in acetonitrile. Red line, cumulative fitted peak; purple lines, independent fitted peaks; black line, raw data.

X-ray photoelectron spectra were taken for unmodified graphite and graphite that had been functionalized by cycling from −0.5 to −2.5 V in the TFAA/pyridine mixture for nine cycles. The functionalized surfaces were additionally sonicated in various solvents to remove unbound material before XPS analysis. Table 1 shows the surface composition of the initial and modified graphite surfaces in terms of the contribution from carbon, nitrogen, oxygen, and fluorine. Figure 3 shows the carbon 1s region of (a) unmodified and (b) functionalized graphite with constituent fitted peaks. Table 2 summarizes the peaks fitted to the experimental data, within the C 1s and N 1s regions, and the area % of each peak, for the modified surface. The full spectra for both modified and unmodified surfaces can be seen in the SI. The survey spectra data (Table 1) show that unmodified graphite did not contain any nitrogen or fluorine, whereas the modified graphite did. In the C 1s region, the experimental data could be fitted with two constituent peaks for the unmodified surface^35,36^ (Figure 3a) corresponding to C–C graphite (284.7 eV) and C–O (286.1 eV) from the reaction of the surface with atmospheric oxygen. These two peaks are not observed for the modified surface (Figure 3b and Table 2); instead, a positive chemical shift is observed, with fitted peaks for carbon environments of pyridine **C**–C–N (285.5 eV) and C–N (286.3 eV), along with C bonded to cationic nitrogen (C–N^+^; 287.8 eV), N–**C**(=O)–CF_3_ (289.2 eV), and CF_3_ (292.7 eV). In the N 1s region, for the modified material, peaks corresponding to neutral N (399 eV) and cationic N (402 eV) were also observed (see the SI).

**Table 1:** Total Atomic % Obtained from the XPS Survey Spectrum for Unmodified and Modified Graphite

	C 1s total at. %	N 1s total at. %	O 1s total at. %	F 1s total at. %
unmodified	93		7	
modified	69	8	8	15

**Table 2:** Fitted XPS Peaks and Associated Areas from the C 1s and N 1s Region of Modified Graphite^a^

binding energy (eV)	285.5	286.3	287.8	289.2	292.7	399.0	402.0
assignment	**C**–C–N	**C**–N	**C**–N^+^	N–**C**=O	**C**F_3_	neutral N	cationic N
area (%)	48	30	11	6	5	48	52

a Bold emphasis shows C species responsible for the peak.

The presence of pyridine on the modified electrode surface is confirmed by the **C**–C–N, **C**–N, and **C**–N^+^ carbon bonding environments. The peak positions of the C=O and CF_3_ environments correspond with those reported for reaction of TFAA with amines to form trifluoroacetamide groups.^37^Table 2 also shows that the N 1s peak fitting is consistent with half the nitrogen environments being neutral and half cationic. Therefore, a plausible structure is a film made up of ∼50% cationic pyridinium salt (**4**) and ∼50% neutral pyridine. One possible mechanism to achieve this would be for some pyridinium species grafted to the electrode surface (or grafted to other pyridinium in the multilayer) to undergo further reduction or hydrolysis, resulting in cleavage of the N–COCF_3_ moiety. Modified electrodes that had been sonicated for 1 h in different solvents to acetonitrile shared the same spectral features as shown in Figure 3b, indicating that the film was strongly grafted to the underlying carbon. Note that in Figure 3b, the data have been fit only with peaks attributed to the film, with no contribution from the underlying graphite. We should be mindful that some contribution from C–C (284.7 eV) and C–O (286.1 eV) from graphite may contribute to the signal but would not be easily resolved from the closely located pyridine C 1s peaks. As discussed in later sections, thinner films result in XPS spectra with resolvable contributions from both graphite and the functionalizing film.

### 3.3 Characterization of the Functionalized Carbon Surface Using Redox Probes

**Figure 4:**
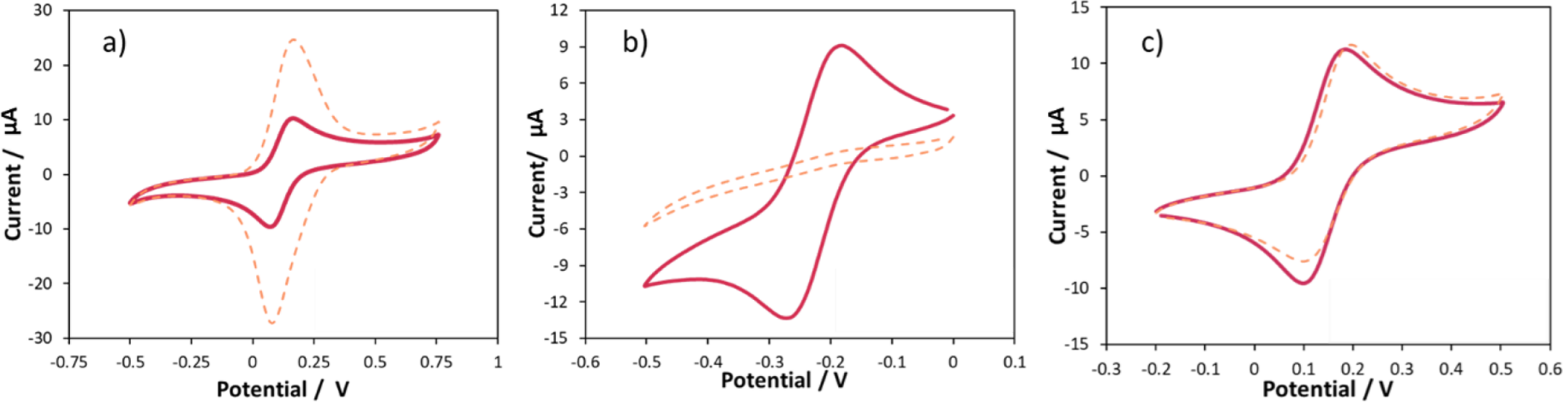
Cyclic voltammograms taken with an unmodified (red) and pyridine/pyridinium-modified (dashed orange) 3 mm-diameter GC electrode. Scan rate of 0.1 V s^–1^; 0.1 M NaCl electrolyte with 1 mM redox probes: (a) ferrocyanide; (b) hexaamineruthenium; (c) ferrocenemethanol. Potentials are reported vs Ag/AgCl.

The nature of the surface layer on a GC electrode was investigated further by determining the interaction with redox probes of different charges (Figure 4), namely, ferrocyanide (Fe(CN)_6_^4–^, negatively charged), hexaamineruthenium (Ru(NH_3_)_6_^3+^, positively charged), and ferrocenemethanol (FcMeOH, neutral). With negatively charged ferrocyanide, redox currents were enhanced at the modified GC compared to the clean electrode (Figure 4a). In contrast, the modified GC in hexaamineruthenium produced no redox peaks, despite a clear redox response being observed for the unmodified electrode (Figure 4b). In FcMeOH, the modified GC oxidation currents matched those of the clean electrode, whereas the reduction currents were slightly decreased (Figure 4c). Similar results were obtained for BDD electrodes that had undergone the same functionalization (see the SI).

These results support the XPS characterization and the proposal that cationic pyridinium moieties contribute significantly to the makeup of the film. Negatively charged ferrocyanide ions are attracted to the positively charged film, resulting in enhanced currents. Moreover, the symmetrical shape of the redox peaks indicates that the redox species are adsorbed and surface-bound and the current is not solely diffusion-controlled. The amount of adsorbed ferrocyanide increases as a function of thickness of the modifying surface layer (see the SI). In hexaamineruthenium, positively charged ions are repelled from the positively charged film, resulting in no redox peaks being observed as the probe cannot reach the electrode surface. For FcMeOH, during oxidation, the neutral molecule undergoes oxidation, and the process is seemingly uninhibited by the presence of the film. It is well-established that the hydrophobic nature of FcMeOH allows it to permeate surface films,^38^ and hence, we see the same current response as at a clean electrode. During the backward scan, the positively charged FcMeOH^+^ undergoes reduction, and the smaller currents for the modified GC again support the repulsion of the positively charged redox probe by the cationic surface film.

### 3.4 Response of Negatively Charged Redox Probes as a Function of the Pyridine Film Protonation State

**Figure 5:**
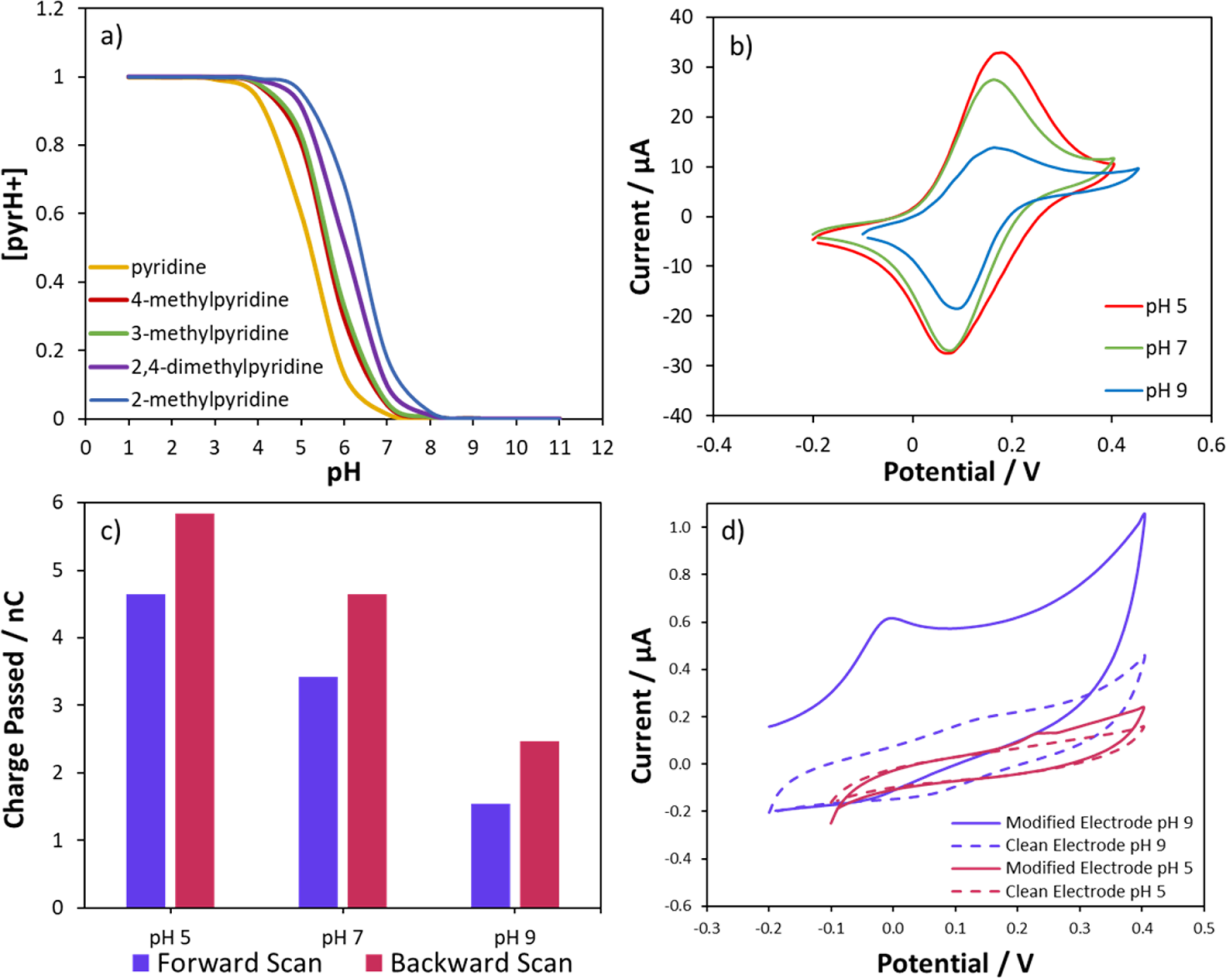
(a) Plot of the calculated relative concentration of protonated pyridine ([pyrH+]) versus the solution pH. [pyrH+] was calculated using the Henderson–Hasselbalch equation. p*K*_a_ values of different substituted pyridines were used as indicated in the legend. (b) CVs (0.1 V s^–1^) of 1 mM ferrocyanide at a functionalized GC electrode at different pH values: red–pH 5, green–pH 7, and blue–pH 9. Potentials are reported vs Ag/AgCl. (c) Charge passed during forward (purple) and backward (red) scans determined by integration of CV peaks. (d) CVs (0.1 V s^–1^) of unmodified GC (dashed) and functionalized GC (undashed) in 0.1 M pH 9 PBS (purple) and 0.1 M pH 5 PBS (red). Potentials are reported vs Ag/AgCl.

The proposed structure of the film, comprising *ca*. 50% cationic trifluoroacetylated pyridinium and 50% neutral pyridine, suggests that although a permanent net positive charge for the layer is expected, the positive charge could be enhanced through protonation of the neutral pyridine moieties. This was assessed by comparing the enhanced current response of the negatively charged ferrocyanide redox probes at different solution pH values. The p*K*_a_ of surface-bound, polymerized pyridine is difficult to determine, but using known p*K*_a_ values for a range of unbound substituted pyridine species,^39^ along with the Henderson–Hasselbalch equation, a plot of the calculated relative concentration of protonated pyridine [pyrH+] as a function of solution pH can be constructed (Figure 5a and the SI). The range of behavior suggests that unbound pyridines are typically 50–95% protonated at pH 5 (depending on substitution), 0–25% protonated at pH 7, and deprotonated at pH 9. To confirm that the modified electrode contained protonatable pyridine functionalities, we therefore investigated the redox response of ferrocyanide at the functionalized GC at pH 5, 7, and 9.

The effect of pH on the CV response of ferrocyanide at the modified electrodes is seen in Figure 5b. Focusing on the forward scan, the largest currents are seen at pH 5 followed closely by pH 7, and then, those at pH 9 are significantly smaller. The peak oxidation current at pH 9 (15 μA) is still higher than that for the unmodified electrode (9 μA, see Figure 4a) indicating a persistent positive charge to the film due to the presence of the cationic trifluoroacetylated pyridinium species. However, the much larger currents at pH 5 and 7 result from significant protonation of the pyridine component, enhancing the positive charge of the film. The current response at pH 7 suggests a greater degree of positive charge than implied by Figure 5a, where 0–25% protonation was predicted. This is not unexpected, as the calculations in Figure 5a used p*K*_a_ values for unbound molecules, while it is well-established that the p*K*_a_ of a surface-bound species is generally several units higher than the unbound molecule and depends strongly on the environment^40^ as well as the applied electrode potential.^41^ Therefore, more than 25% protonation of the surface pyridine groups is certainly feasible at pH 7.

A clear difference can be seen in Figure 5b between the amount of charge passed in the forward and backward scans of the CVs, with larger peak magnitudes on the backward scan. This is particularly clear at pH 9 but is true irrespective of the pH of the solution, as shown in Figure 5c. The response at all pH values is stable to repeated cycling over 10 scans, so the enhanced backward scan current does not seem attributable to time-dependent accumulation of negative species near the surface. The most likely explanation is that direct oxidation of neutral pyridine moieties in the surface film takes place during the forward scan, resulting in a more positively charged surface, which attracts more of the negatively charged redox probe during the subsequent backward scan. The ability of the film to undergo oxidation is confirmed in Figure 5d for the CV of the functionalized GC in pH 9 PBS with no added redox species, where a prominent oxidation peak is seen at about 0 V followed by a rising Faradaic oxidation current up to 0.4 V. This film is believed to be effectively fully deprotonated, so this oxidation response is most likely attributed to neutral pyridine species in the film. Oxidation of such groups would result in the formation of radical cationic species, with the resulting effect of increasing the positive charge of the film. This would therefore explain the enhanced ferricyanide reduction peaks observed, as negatively charged species become attracted to the positive functionalities generated through oxidation of the film during the forward scan. However, the redox response of the film appears irreversible, as there is no reduction peak on the backward scan in Figure 5d. In subsequent scans, the oxidation response is still noted but decreases in magnitude on repeated cycling. Although we observe that the response seen at pH 9 in Figure 5b is stable to repeated cycling, this has been investigated for 10 consecutive CVs only. It is probable that once the film is fully oxidized, the backward peak in Figure 5b may no longer show an enhanced reduction current. A catalytic electron transfer step between the film and solution redox species is another feasible mechanism for the current enhancement but requires further investigation. In contrast, at pH 5, the difference in response at a modified electrode differs little from the clean electrode (Figure 5d), although a small oxidation peak is noted at 0.25 V followed by a rising background. From p*K*_a_ calculations (Figure 5a) and enhanced ferrocyanide response (Figure 5b), we assume that this film is almost fully protonated; we propose therefore that a film composed almost wholly of trifluoroacetylated pyridinium and protonated pyridine has little significant redox response in this potential range.

### 3.5 Toward a Pyridine Multilayer with Reversible Protonation

**Scheme 2:**
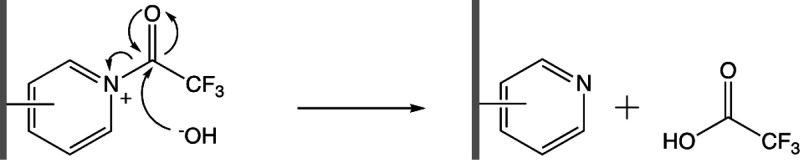
Reaction of Sodium Hydroxide with the Surface-Bound Trifluoroacetylpyridinium to Form Surface-Bound Neutral Pyridine

To enhance the responsiveness of the functionalized carbon surface to protonation, and to allow switching between a fully neutral and positive charge, would require removal of the trifluoroacetyl group from the cationic nitrogens, leaving only neutral pyridine within the film structure. In an attempt to achieve this, the film was treated with a base (1 M NaOH) resulting in pyridine and trifluoroacetic acid/acetate products (Scheme 2). In basic solution, the pyridine groups in such a film should remain deprotonated (neutral), while at pH <5, the films should be close to 100% protonated and hence positively charged.

**Figure 6:**
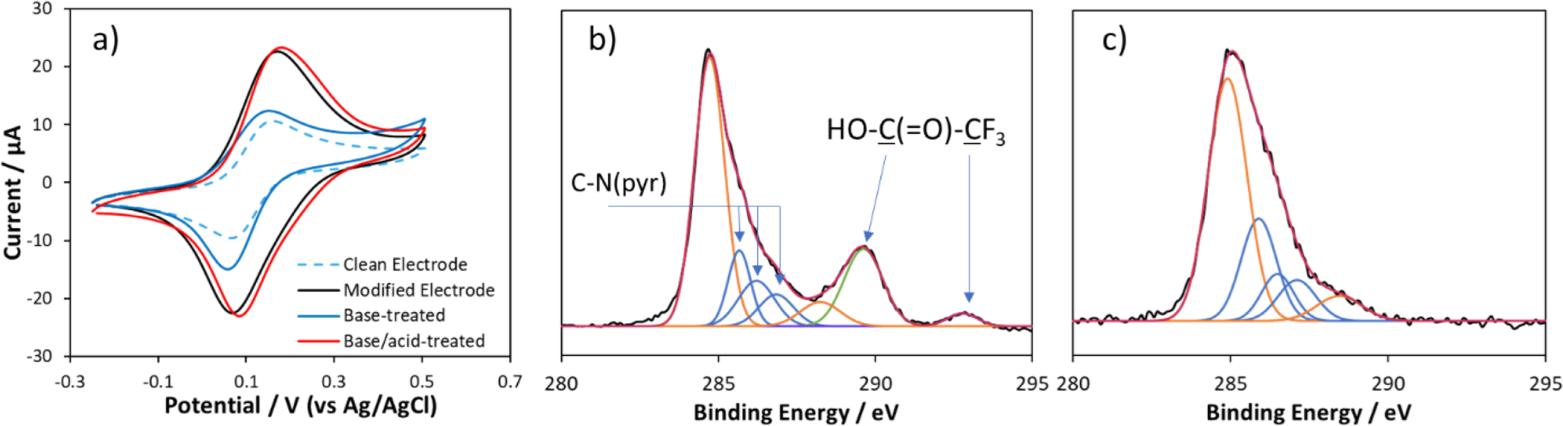
(a) Cyclic voltammograms of 1 mM ferrocyanide in 0.1 M pH 7 PBS with an unmodified GC electrode (dotted blue); GC electrode with pyridine/pyridinium functionalization (black line); functionalized electrode subsequently treated with 1 M NaOH only (blue line) and a functionalized electrode treated with NaOH followed by treatment with 1 M H_2_SO_4_ (red line). (b) Carbon 1s XPS spectrum of functionalized graphite treated with 1 M NaOH. Black line, raw data; red line, cumulative fitted peak; blue lines, independent fitted peaks for pyridine carbon (C–N(pyr)); green lines, fitted peak for trifluoroacetic acid; orange lines, fitted peaks for the underlying graphite surface. (c) Carbon 1s XPS spectrum of functionalized graphite treated with 1 M NaOH and then 1 M H_2_SO_4_. Black line, raw data; red line, cumulative fitted peak; blue lines, independent fitted peak for pyridine carbon (C–N(pyr)); orange lines, fitted peaks for the underlying graphite surface.

To demonstrate the ability to control the protonation state and hence the charge of the surface, CVs of ferrocyanide were recorded at pH 7 (Figure 6 a) with GC modified with (i) the original 50%:50% pyridine and trifluoroacetylpyridinium film (black line), (ii) the film treated with a 1 M NaOH base (solid blue line), and (iii) the film treated with a 1 M NaOH base followed by a 1 M H_2_SO_4_ acid (red line). After base treatment, the dramatically enhanced oxidation currents attributed to the positively charged film are no longer observed; the oxidation current is now much less enhanced compared to the clean, unmodified GC (blue dashed), but the peak is now distinctly diffusion-controlled in shape. In common with the CV recorded at pH 9 in Figure 5b, the reduction peak on the reverse scan is much larger and suggests surface confinement of the redox species. Again, this can be attributed to generation of a positive charge during direct oxidation of the pyridine moieties in the film.

XPS was used to analyze the surface composition of functionalized graphite after base treatment and after base-then-acid treatment (base/acid-treated), the results of which can be seen in Figure 6b,c and Table 3. After base treatment, the C 1s spectrum (Figure 6b) showed peaks for the pyridine carbons (**C**–C–N, **C**–N, and **C**–N^+^, blue) at similar binding energy as those for the untreated film (Figure 3b and Table 2); however, the films examined in Figure 6 were thinner than that shown in Figure 3b; hence, strong contributions from oxidized underlying graphite can be seen (orange peaks: 284.7 eV, C=C; 288.1 eV, C=O). It is believed that the films were thinner as modification was carried out by holding at −2 V for 30 s, rather than by cycling for 9 scans. The spectrum reveals a new peak at 289.7 eV for the base-treated electrode (shown in green), corresponding to the O–C=O carbon environment; this is attributed to liberated trifluoroacetic acid or trifluoroacetate (Scheme 2) trapped in the film due to insufficient rinsing. The continuing presence of −CF_3_ at 292.7 eV supports this interpretation. The contributions from the pyridine carbon in the N 1s spectra also support the loss of some cationic nitrogen on basic treatment, with the 402 eV contribution decreasing from 52% of the peak area to 42%. The persistence of some cationic nitrogen suggests that more prolonged treatment may be required to remove all trifluoroacetyl groups or more likely that some polymerization/coupling via the N (forming C–N^+^–C bonds) may take place during film formation. The fact that the CV response after base treatment shows only a small enhancement, despite the persistence of 42% of cationic N in the structure, indicates that the charged redox species is relatively insensitive to the remaining positive charge of the film. A likely explanation is that the persisting cationic groups are found deeper within the film, while the redox probe interacts with the film-solution interface. In contrast, we believe that XPS probes the full thickness of the film, as we retain some spectral features of the underlying graphite in the XPS spectra of the functionalized surfaces (Figure 6b,c, orange).

**Table 3:** Peak Energy and the Associated Area for Peaks in the N 1s Region of an Electrode Modified with Trifluoroacetylpyridinium (1) and Treated Subsequently with NaOH (2) and Then Further Treated with Sulfuric Acid (3)

		N 1s peak center/eV (area %)	
		399	402
1	untreated	48	52
2	base-treated	58	42
3	base/acid-treated	43	57

After acid treatment, the currents in Figure 6a return to the enhanced values observed for the untreated film at pH 7 (see also Figure 5b). This indicates that, although cationic nitrogen (and hence the positive charge) was removed by the base treatment, after acid treatment, the newly formed surface pyridine groups are now protonated and hence able to attract the ferrocyanide redox species. The net positive surface charge in a pH 7 solution therefore appears the same for the untreated and base-then-acid-treated films, as far as the observed redox behavior is concerned. The advantage of the base treatment is the improved “switchability” of the surface positive charge, with the surface positive charge being removed and replaced by treatment of the electrode in a base and acid, respectively. Thus, this simple base and acid treatment provides the means to maximize the pyridine functionalities in the surface layer, whose charge can be controlled through protonation.

After treatment in an acid (base/acid-treated), both XPS C 1s peaks assigned to the trifluoroacetyl moiety are no longer present (Figure 6 c), confirming that the acetyl group was cleaved and rinsed away. This further supports the probability that the cationic nitrogen remaining in the film after base treatment can be attributed to C–N^+^–C bonds formed through coupling reactions, rather than the remaining pyridinium trifluoroacetyl species. On acid treatment, the amount of cationic nitrogen increases again to 57% according to XPS (Table 3) indicating protonation of a greater number of pyridine functionalities in the film as expected. In principle, one could expect that after treatment in 1 M H_2_SO_4_, all of the nitrogen in the film would be cationic, as all of the pyridine groups would be expected to be protonated at pH <5 (Figure 5a) and the remaining groups are the C–N^+^–C species discussed above. However, the upper limit of 57% cationic N seen in Table 3 likely arises for two reasons: (1) the film is rinsed with deionized water of pH 7 after the base–acid treatment to remove the cleaved groups and sulfate ions; (2) XPS probes the full film thickness, including pyridine within the film that does not undergo protonation if not near the film–solution interface.

### 3.6 Preliminary Screening of Pyridine-Functionalized Carbon for CO_2_ Reduction Activity

Pyridine/pyridinium-functionalized GC, graphite, and BDD electrodes were used to carry out electrochemical CO_2_ reduction, and the solution phase products were determined using NMR. As this was a preliminary screen, no quantification of products was carried out, and gas phase products were not determined. When a potential of −1.2 V vs Ag/AgCl was applied in CO_2_-saturated pH 7.4 PBS, acetone and ethanoic acid were potentially detected as reduction products at the pyridine-functionalized graphite electrode (see the SI), while no reduction product was detected for the unmodified graphite electrode. This preliminary study indicates that such pyridine-functionalized electrodes may be effective CO_2_ reduction electrocatalysts, although significantly more work is needed to quantify the rate of product formation and other potential gas phase products and to determine Faradaic efficiencies. The production of C2 species indicates that C–C bond formation is enhanced on these surfaces, which is consistent with available surface sites for adsorption of CO_2_ and reduction intermediates. The importance of interfacial pH in controlling the reaction mechanism of CO_2_ reduction and the product distribution has been highlighted by both experiment^42^ and theory.^43^ Hence, the ability of the pyridine-functionalized electrode surface to act as a source or sink of protons (effectively acting as a buffer) could potentially allow more targeted product distribution than typically observed on a nonmodified electrode. In addition, the role of amine and pyridine functionalities in complexation and capture of CO_2_ is well-established, suggesting the pyridine functionalization as a means to adsorb and concentrate carbon dioxide and reaction intermediates at the electrode surface.^27^

## 4 Conclusions

Using simple electrochemical reduction of trifluoroacetylpyridinium, carbon surfaces can be functionalized with layers composed of linked pyridinium and pyridine moieties at room temperature, on a timescale of minutes. As-prepared films have a net positive charge in solution due to the pyridinium content, and this can be enhanced further through protonation of the neutral pyridine component by controlling the solution pH. Moreover, the nitrogen–acetyl bond can be cleaved through base treatment to purposefully increase the neutral pyridine proportion of the film. This results in a surface that can be “switched” from nearly neutral to a positive charge by treatment in basic and acidic solutions, respectively, through manipulation of the protonation state of the pyridine.

Such functionalized surfaces present a means to test in isolation the specific catalytic performance of pyridinic groups toward key processes such as oxygen and CO_2_ reduction. The functionalization process demonstrated here is achievable under room-temperature conditions using stable precursors and hence can allow for rapid screening of surface properties. Using different precursors added to TFAA to form a salt, we should, in principle, be able to modify carbon surfaces with a range of heteroaromatic species, including pyrimidines, conjugated pyridines (such as quinoline or isoquinoline), and imidazoles. This would allow the properties of such functionalized surfaces to be systematically investigated in a way that is less conveniently achieved using other synthetic routes to doped carbons.

## Supporting Information Available

NMR analysis of compounds **1**–**3**; CV responses of **1** and **2**; addition of a styrene radical trap; calculation of thickness of the modifying layer; functionalization of other electrodes and use of different pyridine precursors; details of computational calculations; full XPS spectra for modified electrodes and those treated with an acid and base; p*K*_a_ calculations using the Henderson–Hasselbalch equation; preliminary NMR product analysis of CO_2_ reduction using pyridine-modified electrodes (PDF)
DFT calculations carried out using the Gaussian-16 software package (ZIP)

## References

[ref1] Guo D.; Shibuya R.; Akiba C.; Saji S.; Kondo T.; Nakamura J. Active sites of nitrogen-doped carbon materials for oxygen reduction reaction clarified using model catalysts. Science 2016, 351, 361–365. 10.1126/science.aad0832. 26798009

[ref2] Liu S.; Yang H.; Huang X.; Liu L.; Cai W.; Gao J.; Li X.; Zhang T.; Huang Y.; Liu B. Identifying Active Sites of Nitrogen-Doped Carbon Materials for the CO_2_ Reduction Reaction. Adv. Funct. Mater. 2018, 28, 1800499 10.1002/adfm.201800499.

[ref3] Guo W.; Li X.; Xu J.; Liu H. K.; Ma J.; Dou S. X. Growth of Highly Nitrogen-Doped Amorphous Carbon for Lithium-ion Battery Anode. Electrochim. Acta 2016, 188, 414–420. 10.1016/j.electacta.2015.12.045.

[ref4] Prathish K. P.; Barsan M. M.; Geng D.; Sun X.; Brett C. M. A. Chemically modified graphene and nitrogen-doped graphene: Electrochemical characterisation and sensing applications. Electrochim. Acta 2013, 114, 533–542. 10.1016/j.electacta.2013.10.080.

[ref5] Cai J.; Wu C.; Zhu Y.; Zhang K.; Shen P. K. Sulfur impregnated N, P co-doped hierarchical porous carbon as cathode for high performance Li-S batteries. J. Power Sources 2017, 341, 165–174. 10.1016/j.jpowsour.2016.12.008.

[ref6] Shao Y.; Zhang S.; Engelhard M. H.; Li G.; Shao G.; Wang Y.; Liu J.; Aksay I. A.; Lin Y. Nitrogen-doped graphene and its electrochemical applications. J. Mater. Chem. 2010, 20, 7491–7496. 10.1039/c0jm00782j.

[ref7] Gong K.; Du F.; Xia Z.; Durstock M.; Dai L. Nitrogen-Doped Carbon Nanotube Arrays with High Electrocatalytic Activity for Oxygen Reduction. Science 2009, 323, 760–764. 10.1126/science.1168049. 19197058

[ref8] Li B.; Dai F.; Xiao Q.; Yang L.; Shen J.; Zhang C.; Cai M. Nitrogen-doped activated carbon for a high energy hybrid supercapacitor. Energy Environ. Sci. 2016, 9, 102–106. 10.1039/C5EE03149D.

[ref9] Salvador G. P.; Gerosa M.; Sacco A.; Garido N.; Castelline M.; Massaglia G.; Delmondo L.; Agostino V.; Margaria V.; Chiodoni A. Green-Synthesized Nitrogen-Doped Carbon-Based Aerogels as Environmentally Friendly Catalysts for Oxygen Reduction in Microbial Fuel Cells. Energy Technol. 2018, 6, 1052–1059. 10.1002/ente.201700615.

[ref10] Gross A. J.; Downard A. J. Regeneration of Pyrolyzed Photoresist Film by Heat Treatment. Anal. Chem. 2011, 83, 2397–2402. 10.1021/ac103264v. 21344943

[ref11] Öztürk A.; Yurtcan A. B. Preparation and characterization of melamine-led nitrogen-doped carbon blacks at different pyrolysis temperatures. J. Solid State Chem. 2021, 296, 121972 10.1016/j.jssc.2021.121972.

[ref12] Singh S. K.; Takeyasu K.; Nakamura J. Active Sites and Mechanism of Oxygen Reduction Reaction Electrocatalysis on Nitrogen-Doped Carbon Materials. Adv. Mater. 2019, 31, 1804297 10.1002/adma.201804297. 30350433

[ref13] Reddy A. L. M.; Srivastava A.; Gowda S. R.; Gullapalli H.; Dudey M.; Ajayan P. M. Synthesis Of Nitrogen-Doped Graphene Films For Lithium Battery Application. ACS Nano 2010, 4, 6337–6342. 10.1021/nn101926g. 20931996

[ref14] Ćirić-Marjonović G.; Pašti I.; Gavrilov N.; Janošević A.; Mentus S. Carbonised polyaniline and polypyrrole: towards advanced nitrogen-containing carbon materials. Chem. Pap. 2013, 67, 781–813. 10.2478/s11696-013-0312-1.

[ref15] Su Y.; Liu S.; Ye G.; Zhu W.; Zhao K.; Huang R.; He Z. ZnCl_2_ as a “Nitrogen Bank” to Inhibit Nitrogen Loss during the Thermal Conversion of Nitrogen-Containing Carbon Precursors to Nitrogen-Doped Carbon. ACS Appl. Energy Mater. 2021, 4, 5375–5380. 10.1021/acsaem.1c00689.

[ref16] Nan Y.; He Y.; Zhang Z.; Wei J.; Zhang Y. Controllable synthesis of N-doped carbon nanohorns: tip from closed to half-closed, used as efficient electrocatalysts for oxygen evolution reaction. RSC Adv. 2021, 11, 35463–35471. 10.1039/D1RA06458D. 35493191 PMC9043249

[ref17] Lv Q.; Si W.; He J.; Sun L.; Zhang C.; Wang N.; Yang Z.; Li X.; Wang X.; Deng W.; Long Y.; Huang C.; Li Y. Selectively nitrogen-doped carbon materials as superior metal-free catalysts for oxygen reduction. Nat. Commun. 2018, 9, 3376 10.1038/s41467-018-05878-y. 30139938 PMC6107639

[ref18] Tuci G.; Zafferoni C.; D’Ambrosio P.; Caporali S.; Ceppatelli M.; Rossin A.; Tsoufis T.; Innocenti M.; Giambastiani G. Tailoring Carbon Nanotube N-Dopants while Designing Metal-Free Electrocatalysts for the Oxygen Reduction Reaction in Alkaline Medium. ACS Catal. 2013, 3, 2108–2111. 10.1021/cs400379h.

[ref19] Tuci G.; Zafferoni C.; Rossin A.; Milella A.; Luconi L.; Innocenti M.; Phuoc L. T.; Duong-Viet C.; Pham-Huu C.; Giambastiani G. Chemically Functionalized Carbon Nanotubes with Pyridine Groups as Easily Tunable N-Decorated Nanomaterials for the Oxygen Reduction Reaction in Alkaline Medium. Chem. Mater. 2014, 26, 3460–3470. 10.1021/cm500805c.

[ref20] Bayazit M. K.; Clarke L. S.; Coleman K. S.; Clarke N. Pyridine-Functionalized Single-Walled Carbon Nanotubes as Gelators for Poly(acrylic acid) Hydrogels. J. Am. Chem. Soc. 2010, 132, 15814–15819. 10.1021/ja1076662. 20945903

[ref21] Agullo J.; Canesi S.; Schaper F.; Morin M.; Bélanger D. Formation and Reactivity of 3-Diazopyridinium Cations and Influence on Their Reductive Electrografting on Glassy Carbon. Langmuir 2012, 28, 4889–4895. 10.1021/la2048757. 22324405

[ref22] Smida H.; Lebèrge E.; Bergamini J.-F.; Barrière F.; Lagrost C. Reductive electrografting of in situ produced diazopyridinium cations: Tailoring the interface between carbon electrodes and electroactive bacterial films. Bioelectrochemistry 2018, 120, 157–165. 10.1016/j.bioelechem.2017.12.006. 29275091

[ref23] Agullo J.; Morin M.; Bélanger D. Modification of Glassy Carbon Electrode by Electrografting of In Situ Generated 3-diazopyridinium Cations. J. Electrochem. Soc. 2012, 159, H758–H764. 10.1149/2.054209jes.

[ref24] Haziri V.; Berisha A.; Podvorica F. I. Electrochemical modification of platinum and glassy carbon surfaces with pyridine layers and their use as complexing agents for copper (II) ions. Open Chem. 2019, 17, 277–727. 10.1515/chem-2019-0084.

[ref25] Li Q.; Schönleber K.; Zeller P.; Höhlen I.; Rieger B.; Wintterlin J.; Krischer K. Activation of silicon surfaces for H_2_ evolution by electrografting of pyridine molecules. Surface Sci. 2015, 631, 185–189. 10.1016/j.susc.2014.07.007.

[ref26] Yeşildağ A.; Ekinci D. Covalent attachment of pyridine-type molecules to glassy carbon surfaces by electrochemical reduction of in situ generated diazonium salts. Formation of ruthenium complexes on ligand-modified surfaces. Electrochim. Acta 2010, 55, 7000–7009. 10.1016/j.electacta.2010.06.020.

[ref27] Bae Y. -S.; Liu J.; Wilmer C. E.; Sun H.; Dickey A. N.; Kim M. B.; Benin A. I.; Willis R. R.; Barpaga D.; LeVan M. D.; Snurr R. Q. The effect of pyridine modification of Ni–DOBDC on CO_2_ capture under humid conditions. Chem. Commun. 2014, 50, 3296–3298. 10.1039/C3CC44954H. 24527490

[ref28] Samata S. K.; Dey N.; Kumari N.; Biswakarma D.; Bhattachara S. Multimodal Ion Sensing by Structurally Simple Pyridine-End Oligo p-Phenylenevinylenes for Sustainable Detection of Toxic Industrial Waste. ACS Sustainable Chem. Eng. 2019, 7, 12304–12314. 10.1021/acssuschemeng.9b01644.

[ref29] Frisch M. J.; Trucks G. W.; Schlegel H. B.; Scuseria G. E.; Robb M. A.; Cheeseman J. R.; Scalmani G.; Barone V.; Petersson G. A.; Nakatsuji H.; Li X.; Caricato M.; Marenich A. V.; Bloino J.; Janesko B. G.; Gomperts R.; Mennucci B.; Hratchian H. P.; Ortiz J. V.; Izmaylov A. F.; Sonnenberg J. L.; Williams-Young D.; Ding F.; Lipparini F.; Egidi F.; Goings J.; Peng B.; Petrone A.; Henderson T.; Ranasinghe D.; Zakrzewski V. G.; Gao J.; Rega N.; Zheng G.; Liang W.; Hada M.; Ehara M.; Toyota K.; Fukuda R.; Hasegawa J.; Ishida M.; Nakajima T.; Honda Y.; Kitao O.; Nakai H.; Vreven T.; Throssell K.; Montgomery J. A. Jr.; Peralta J. E.; Ogliaro F.; Bearpark M. J.; Heyd J. J.; Brothers E. N.; Kudin K. N.; Staroverov V. N.; Keith T. A.; Kobayashi R.; Normand J.; Bloino J.; Janesko B. G.; Gomperts R.; Mennucci B.; Hratchian H. P.; Ortiz J. V.; Izmaylov A. F.; Sonnenberg J. L.; Williams-Young D.; Ding F.; Lipparini F.; Egidi F.; Goings J.; Peng B.; Petrone A.; Henderson T.; Ranasinghe D.; Zakrzewski V. G.; Gao J.; Rega N.; Zheng G.; Liang W.; Hada M.; Ehara M.; Toyota K.; Fukuda R.; Hasegawa J.; Ishida M.; Nakajima T.; Honda Y.; Kitao O.; Nakai H.; Vreven T.; Throssell K.; Montgomery J. A. Jr.; Peralta J. E.; Ogliaro F.; Bearpark M. J.; Heyd J. J.; Brothers E. N.; Kudin K. N.; Staroverov V. N.; Keith T. A.; Kobayashi R.; Normand J.; Raghavachari K.; Rendell A. P.; Burant J. C.; Iyengar S. S.; Tomasi J.; Cossi M.; Millam J. M.; Klene M.; Adamo C.; Cammi R.; Ochterski J. W.; Martin R. L.; Morokuma K.; Farkas O.; Foresman J. B.; Fox D. J. Gaussian 16, Revision C.01, Gaussian, Inc.: Wallingford CT, 2019.

[ref30] Roth H. G.; Romero N. A.; Nicewicz D. A. Experimental and Calculated Electrochemical Potentials of Common Organic Molecules for Applications to Single-Electron Redox Chemistry. Synlett 2016, 27, 714–723. 10.1055/s-0035-1561297.

[ref31] Isse A. A.; Gennaro A. Absolute potential of the standard hydrogen electrode and the problem of interconversion of potentials in different solvents. J. Phys. Chem. B 2010, 114, 7894–7899. 10.1021/jp100402x. 20496903

[ref32] Pavlishchuk V. V.; Addison A. W. Conversion constants for redox potentials measured versus different reference electrodes in acetonitrile solutions at 25°C. Inorg. Chim. Acta 2000, 298, 97–102. 10.1016/S0020-1693(99)00407-7.

[ref33] Lu T.; Chen F. Multiwfn: A multifunctional wavefunction analyzer. J. Comput. Chem. 2012, 33, 580–592. 10.1002/jcc.22885. 22162017

[ref34] Becke A. D. A multicenter numerical integration scheme for polyatomic molecules. J. Chem. Phys. 1988, 88, 2547–2553. 10.1063/1.454033.

[ref35] Gengenbach T. R.; Major G. H.; Linford M. R.; Easton C. D. Practical guides for x-ray photoelectron spectroscopy (XPS): Interpreting the carbon 1s spectrum. J. Vac. Sci. Technol., A 2021, 39, 013204 10.1116/6.0000682.

[ref36] Beamson G.; Briggs D. High Resolution XPS of Organic Polymers the Scienta ESCA300 Database, Wiley: Chichester, 1992.

[ref37] Pippig F.; Sarghini S.; Hollander A.; Paulussen S.; Terryn H. TFAA chemical derivatization and XPS. Analysis of OH and NHx polymers. Surf. Interface Anal. 2009, 41, 421–429. 10.1002/sia.3043.

[ref38] Etienne M.; Quach A.; Grosso D.; Nicole L.; Sanchez C.; Walcarius A. Molecular Transport into Mesostructured Silica Thin Films: Electrochemical Monitoring and Comparison between p6m, P63/mmc, and Pm3n Structures. Chem. Mater. 2007, 19, 844–856. 10.1021/cm0625068.

[ref39] Mech P.; Bogunia M.; Nowacki A.; Makowski M. Calculations of pKa Values of Selected Pyridinium and Its N-Oxide Ions in Water and Acetonitrile. J. Phys. Chem. A 2020, 124, 538–551. 10.1021/acs.jpca.9b10319. 31856569

[ref40] Lounasvuori M. M.; Holt K. B. Acid deprotonation driven by cation migration at biased graphene nanoflake electrodes. Chem. Commun. 2017, 53, 2351–2354. 10.1039/C6CC09418J. 28164180

[ref41] Burgess I.; Seivewright B.; Lennox R. B. Electric field driven protonation/deprotonation of self-assembled monolayers of acid-terminated thiols. Langmuir 2006, 22, 4420–4428. 10.1021/la052767g. 16618197

[ref42] Schouten K. J. P.; Gallant E. P.; Koper M. T. M. The influence of pH on the reduction of CO and CO_2_ to hydrocarbons on copper electrodes. J. Electroanal. Chem. 2014, 716, 53–57. 10.1016/j.jelechem.2013.08.033.

[ref43] Liu X.; Schlexer P.; Xiao J.; Ji Y.; Wang L.; Sandberg R. B.; Tang M.; Brown K. S.; Peng H.; Ringe S.; Hahn C.; Jaramillo T. F.; Nørskov J.; Chan K. pH effects on the electrochemical reduction of CO_(2)_ towards C_2_ products on stepped copper. Nat. Commun. 2019, 10, 32 10.1038/s41467-018-07970-9. 30604776 PMC6318338

