# Supplementary material for: Room-Temperature One-Pot Synthesis of pH-Responsive Pyridine-Functionalized Carbon Surfaces
Source: ACS Omega. 2023 Mar 17;8(12):10796–805. doi: 10.1021/acsomega.2c06847 (PMC10061597; doi:10.1021/acsomega.2c06847)
Supplement: Supplementary file 1 — ao2c06847_si_001.pdf [file ao2c06847_si_001.pdf]

Supporting Information
Room temperature one-pot synthesis of pH responsive pyridine-functionalised carbon surfaces

Isobel M. Wilson, Sandeep K. Padamati, Antonia D. Bobitan,
Michael J. Porter and Katherine B. Holt*
Department of Chemistry, University College London,
20 Gordon St, London, WC1H 0AJ, UK

Contents:

1: NMR Analysis of solutions containing Pyridine, TFAA and TFAA+Pyridine

2: CV response of TFAA and pyridine

3: Additional of styrene radical trap during reduction of **3**

4: Calculation of thickness of pyridine / pyridinium layer

5: CVs for functionalisation of graphite rods and BDD and gold and Pt

6: Modification of carbon surface with other substituted pyridines.

7: Calculation of redox potentials.

8: Full XPS spectra of non-modified graphite and pyridine-functionalised graphite.

9: Current enhancement / surface adsorption of ferrocyanide as a function of surface modification

10: Response of pyridine-modified boron doped diamond (BDD) to different redox probes

11: Example of use of Henderson Hasselbalch equation to determine [pyrH+] as a function of pH

12: XPS of Modified Electrode Treated with NaOH

13: XPS of Modified Electrode Treated with H_2SO_4

14: CO_2 reduction using pyridine modified carbon electrodes – preliminary solution product analysis

1: NMR Analysis of solutions containing Pyridine, TFAA and TFAA+Pyridine

Table S1: Proton NMR analysis of solutions containing pyridine and TFAA+pyridine mixture.

Species	Proton NMR Shifts (ppm)				
	H_{pyr-2}	H_{pyr-3}	H_{pyr-4}	H_{pyr-5}	H_{pyr-6}
Pyridine	8.56	7.32	7.72	-	-
TFAA + Pyridine	8.79	7.88	8.39	-	-

Table S2: Carbon NMR analysis of solutions containing TFAA alone, pyridine alone and TFAA+pyridine mixture.

Species	Carbon NMR Shifts (ppm)						
	C_{pyr-2}	C_{pyr-3}	C_{pyr-4}	C_{pyr-5}	C_{pyr-6}	C=O	CF_3
TFAA						158.3	115.8
Pyridine	150.8	124.8	136.9	-	-		
TFAA + Pyridine	143.9	127.2	145.2	-	-	162.3	118.0

2. CV responses of TFAA and pyridine

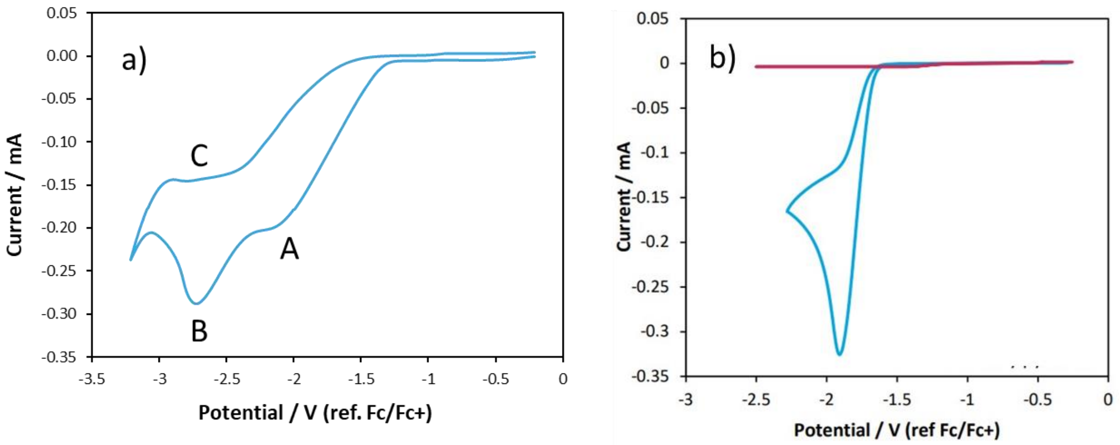

Figure S1: Cyclic voltammograms for the reduction of a) 15 mM TFAA and b) 10 mM pyridine alone (red) and mixture of 15 mM TFAA + 10 mM pyridine (blue). Glassy carbon (GC) working electrode, scan rate 0.1 V s^{-1}, electrolyte 0.1 M $TBAPF_6$ in acetonitrile.

Cyclic voltammograms (CVs) were measured for TFAA (**1**) (Figure S1a), where during initial reduction at -2.15 V (A), we propose the TFAA is reduced to produce a trifluoroacetyl radical (**2**) and a carboxylate. A second reduction step at -2.70 V (B) is attributed to reduction of the radical (**2**) to the acyl anion (**3**). See scheme 1 in main manuscript. The sharp shape of peak B suggests some electrode passivation also takes place, possibly due to reaction of interfacial trifluoroacetyl radicals with the electrode. Such passivation appears temporary, as a reduction current (C) is evident on the backward scan, showing that **1** and **2** remaining in the diffusion layer can be reduced on the return sweep once the adsorbed layer is removed. Repeated consecutive CVs of TFAA are similar to those in Fig S1 a, showing no permanent passivation of the electrode surface by TFAA reduction products.

CVs were measured for 10 mM pyridine in 0.1 M TBAPF6 at a GC electrode, as shown in Fig S1 b (red). The CV is featureless in the region scanned, showing that it is not possible to reduce pyridine in this potential range. In contrast, on addition of 15 mM TFAA to the solution as new redox response is noted (blue) which is attributed to the *in situ* generation of the trifluoroacetylpyridinium salt (see main text).

3. Addition of styrene radical trap during reduction of 3

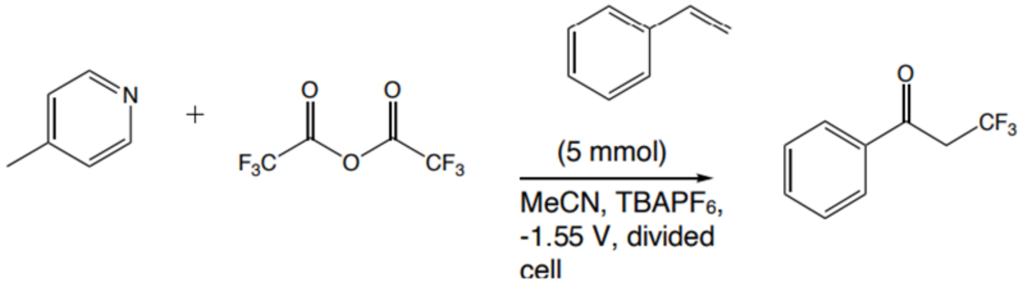

Scheme S1: Addition of styrene to the TFAA+pyridine mixture during reaction at -1.55 V results in formation of fluorinated ketone product at up to 50% yield (depending on reaction conditions) as determined by fluorine NMR.

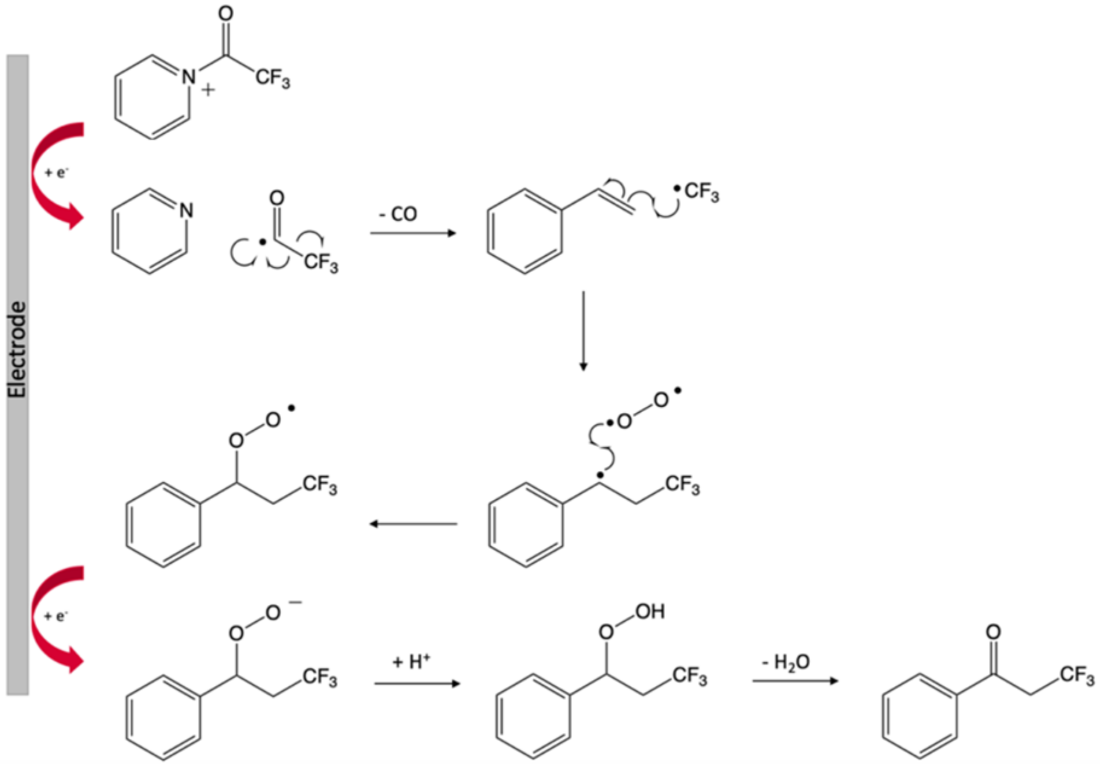

Scheme S2: Proposed mechanism for formation of fluorinated ketone product by electrochemical reduction of 3 with addition of styrene. Key first step is reduction of 3 to form the radical species shown in computational calculations (see main text Fig 2). Radical can either graft to electrode surface or undergo rearrangement to give free pyridine, CO and CF_3 radical.

4. Calculation of thickness of pyridine / pyridinium layer

Total charge passed over 9 consecutive voltammograms = -0.16 mC

(Determined by integration of CV reduction peaks).

Divide the total charge by the charge of one electron:

$$-0.16 \text{ x } 10^{-3} / 1.6 \text{ x } 10^{-19} = 1 \text{ x } 10^{15}$$

If we assume that 2 electrons are required for the reduction of each pyridinium molecule: $0.5 \text{ x } 10^{15}$ pyridinium molecules were reduced and grafted to the electrode surface.

Estimated radius of a pyridinium molecule (based on values from *Electrophoresis* 1994, 15, 635-639) = 3.115 Å

Area of a pyridinium molecule in Å^2: $\pi \, x \, 3.115^2 = 30.5$

Area of pyridinium molecule in mm^2 = $3.05 \text{ x } 10^{-13}$ mm^2

Area of electrode surface = 50.27 mm^2

Number of pyridinium molecules that can fit on electrode surface:

$$50.27 / 3.05 \, x \, 10^{-13} = 1.705 \, x \, 10^{14}$$

Divide the total number of pyridinium molecules reduced by the number of pyridinium molecules per layer:

$$0.5 \, x \, 10^{15} / 1.705 \, x \, 10^{14} = 2.85$$

Estimated number of layers in film = 2.85.

5. CVs for functionalisation of graphite rods and BDD and gold and Pt

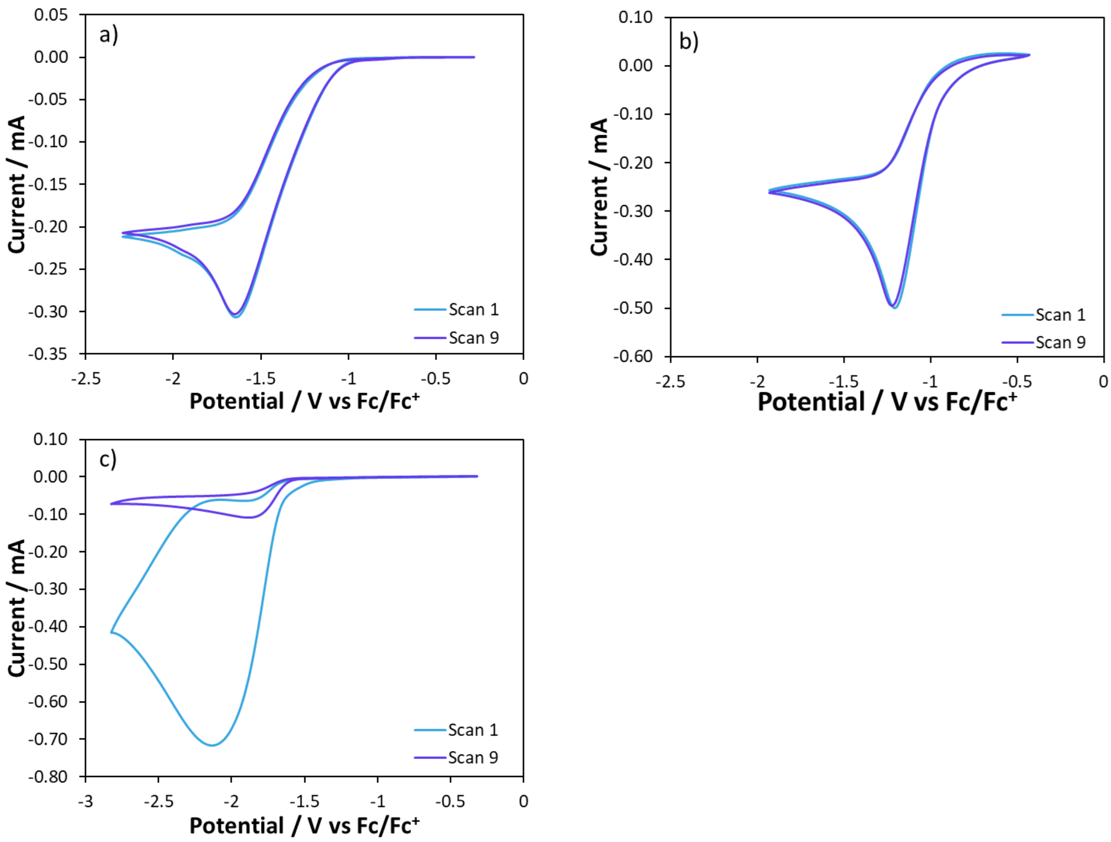

Figure S2: Cyclic voltammograms of the 1st and 9th scans of a 10 mM solution of trifluoroacetylpyridinium in degassed MeCN with $TBAPF_6$ supporting electrolyte, scan rate 0.1 V s^{-1} with different working electrodes: a) gold; b) platinum and c) boron doped diamond

No passivation of current response is seen for gold or Pt indicating that surface grafting / functionalisation with pyridine / pyridinium does not take place. In contrast, at the boron-doped diamond (BDD) electrode the current decreases with consecutive scans, showing formation of the functionalising layer. The current is not as suppressed by scan 9 as observed on GC or graphite, indicating that the surface layer may be less dense and thick on the BDD surface due to its less reactive nature.

6: Modification of carbon surface with other substituted pyridines.

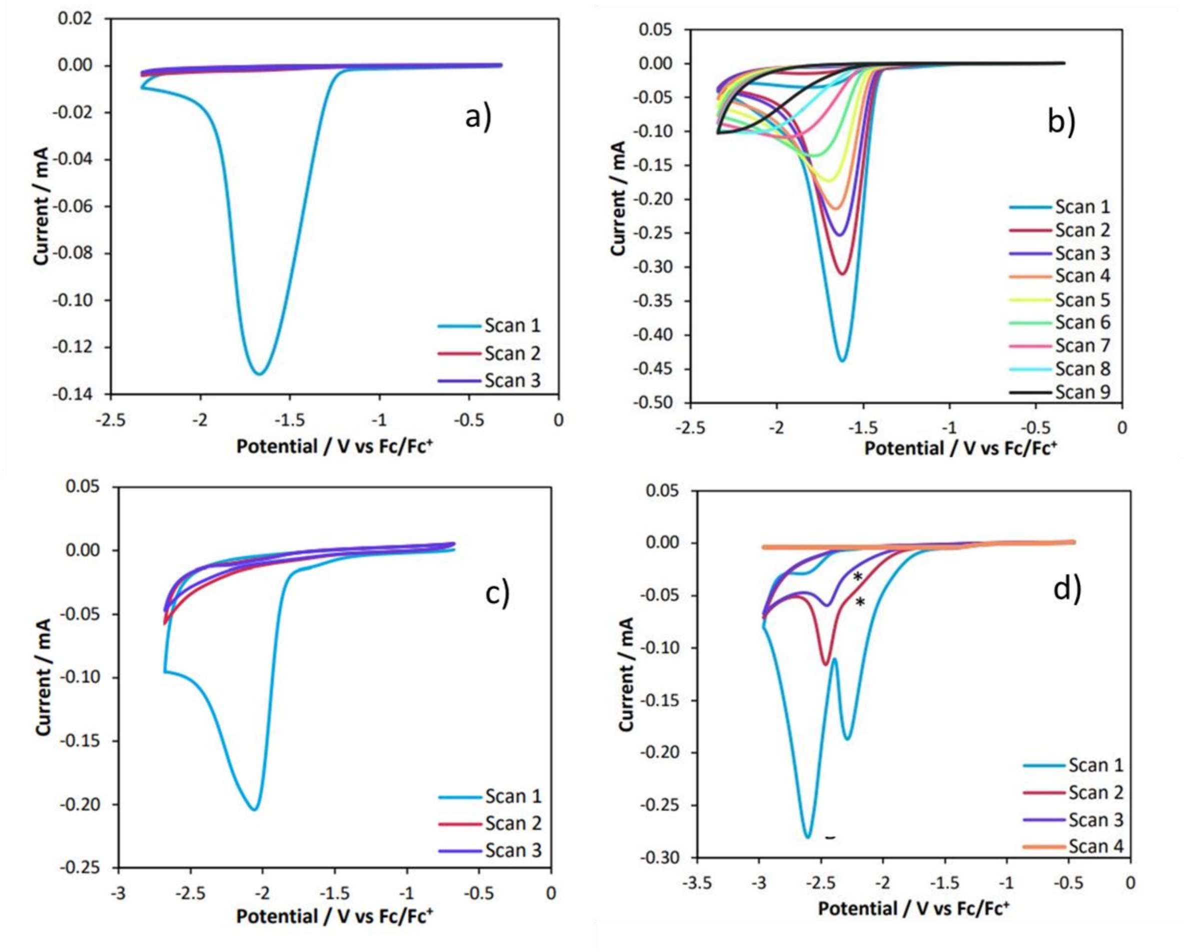

Figure S3: CVs Glassy carbon (GC) working electrode, scan rate 0.1 V s^{-1}, electrolyte 0.1 M $TBAPF_6$ in acetonitrile with 15 mM TFAA and a) 10 mM 2-iodopyridine; b) 10 mM 3-bromopyridine; c) 10 mM 4-bromo-2-methylpyridine and d) 10 mM 4-dimethylaminopyridine.

Fig S3 shows the CV response for mixtures of TFAA and different substituted pyridine species. As for the unsubstituted pyridine a new redox response is seen for the mixtures, indicating the in situ generation of the substituted pyridinium salt which can undergo reduction. All species show passivation of the response over a varying number of scans. Functionalisation of the GC surface with pyridine / pyridinium films was confirmed in each case by XPS (not shown). The thickness / density of the films and specific surface speciation vary as a function of the substitution of the pyridine species. These are preliminary results that require further analysis and investigation.

7. Calculation of redox potentials

Table S3: Calculated free energies for structures subjected to geometry optimisation in their oxidised and reduced forms at the B3LYP/6-31+G(d,p) level with CPCM solvation.

Compound	Charge	G^{calc}/Hartree	$E^{0,\text{calc}}$/V
TFAA ($CF_3COOCOCF_3$)	0	–977.178728	–1.31
TFAA ($CF_3COOCOCF_3$)	–1	–977.306971	
Pyridine (C_5H_5N)	0	–248.247640	–3.15
Pyridine (C_5H_5N)	–1	–248.308171	
N-Trifluoroacetylpyridine ($CF_3CONC_5H_5$)	+1	–699.019732	–0.19
N-Trifluoroacetylpyridine ($CF_3CONC_5H_5$)	0	–699.189220	

Redox potentials were calculated according to the equation

$$E^{0,\text{calc}} = -\frac{(G_{298}^{\text{red}} - G_{298}^{\text{ox}})}{F} - 4.802\ \text{V}$$

Example:

For TFAA, $G_{298}^{\text{red}} - G_{298}^{\text{ox}}$ = [–977.306971 – (–977.178728)] Hartree

= –0.12824 Hartree

= –336.70 kJ mol^{-1}

Hence $E^{0,\text{calc}} = -\frac{-336700}{96485} - 4.802$ V = (3.490 – 4.802) V

= –1.31 V

Coordinate files for all optimised structures are available in MOL format as a zipped ESI file.

8. Full XPS spectra of non-modified graphite and pyridine-modified graphite

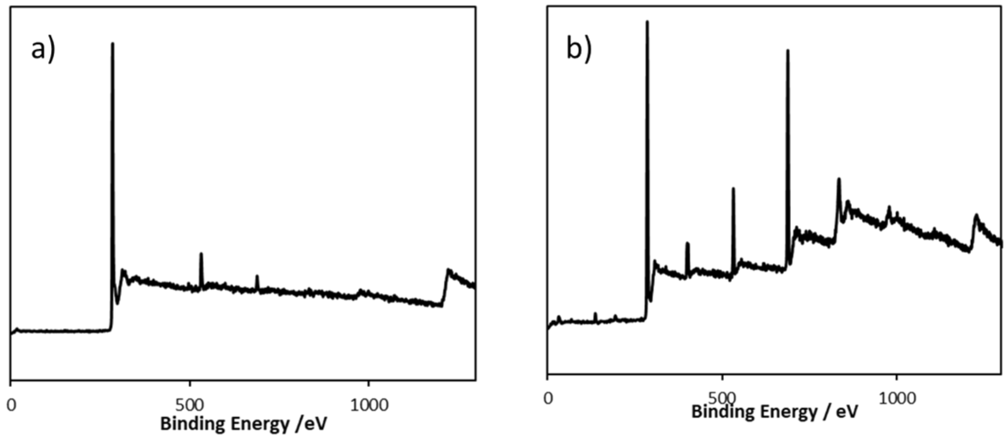

Figure S4: XPS Survey scans of a) unmodified graphite and b) graphite functionalised by electrochemical reduction of trifluoroacetylpyridinium salt.

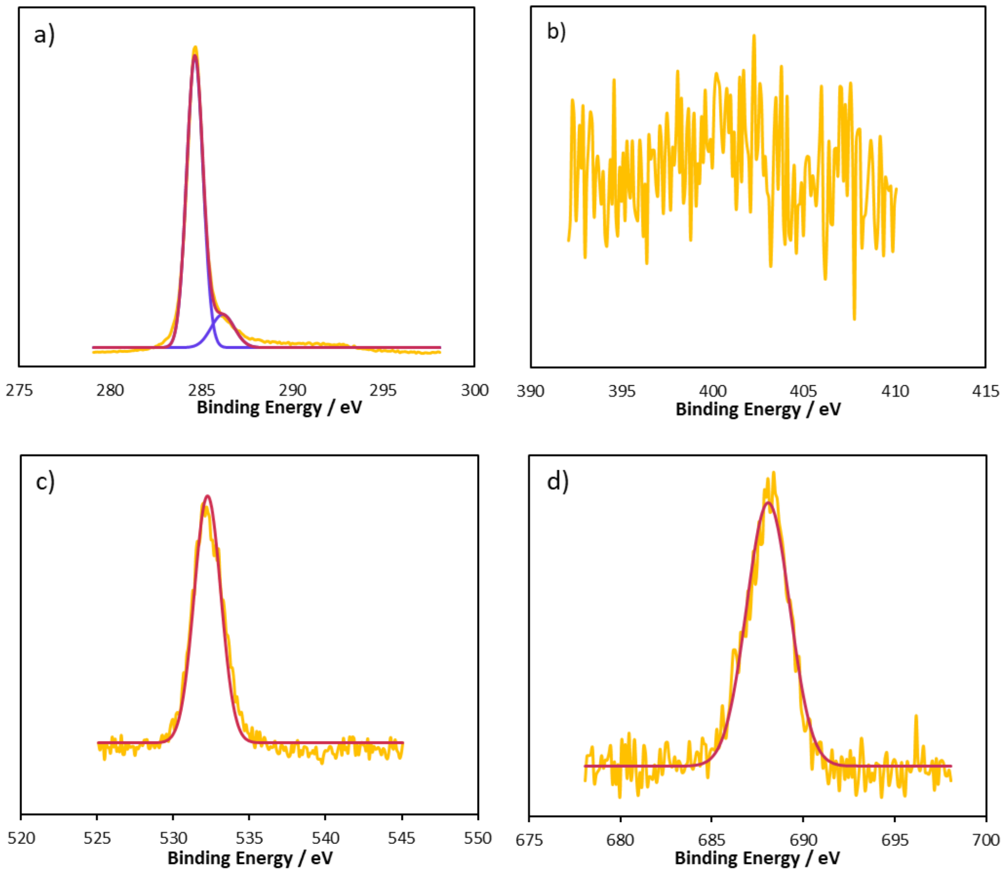

Figure S5: XPS data of a clean carbon electrode showing the a) C1s region; b) N1s region; c) O1s region; d) F1s region; Red line – cumulative fit; blue line – individual peaks; yellow line – raw data.

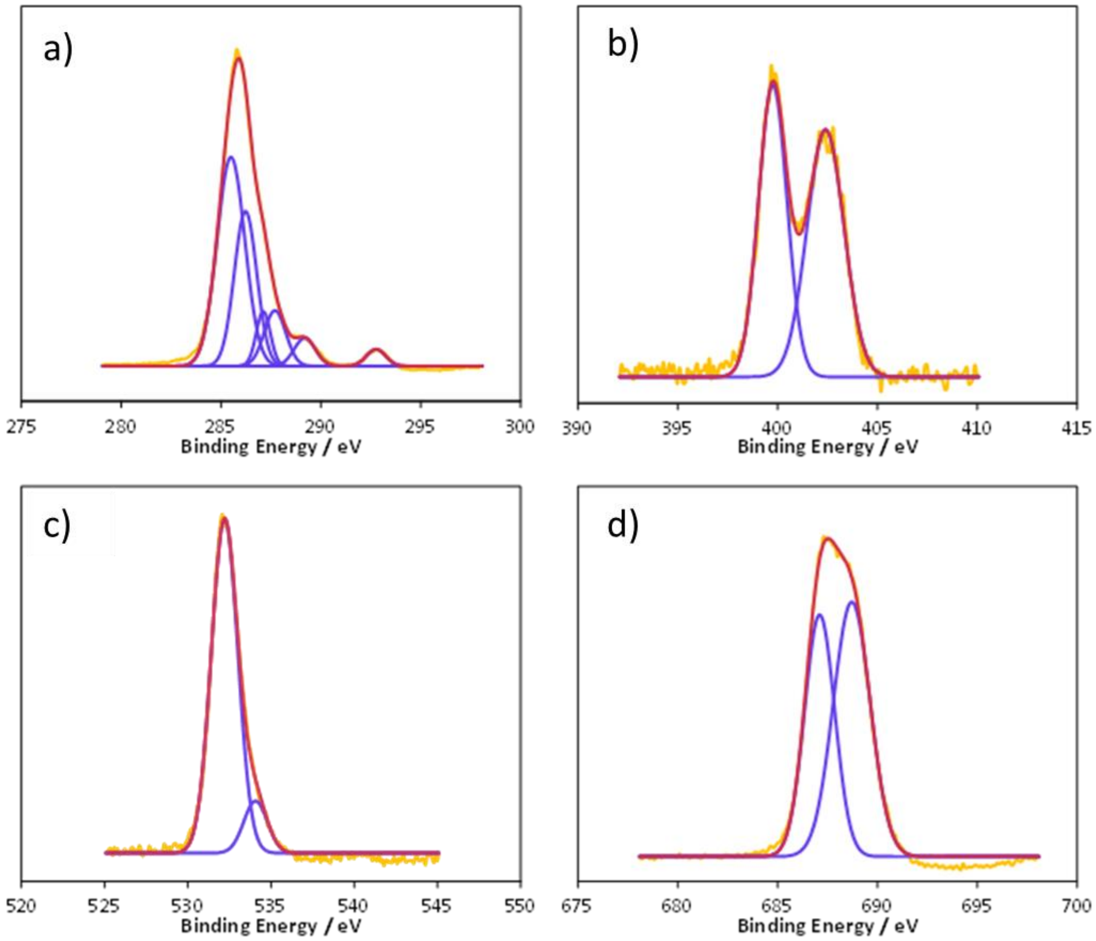

Figure S6: XPS data of graphite functionalised by electrochemical reduction of trifluoroacetylpyridinium salt showing the a) C1s region; b) N1s region; c) O1s region; d) F1s region; Red line – cumulative fit; blue line – individual peaks; yellow line – raw data.

9. Current enhancement / surface adsorption of ferrocyanide as a function of surface modification

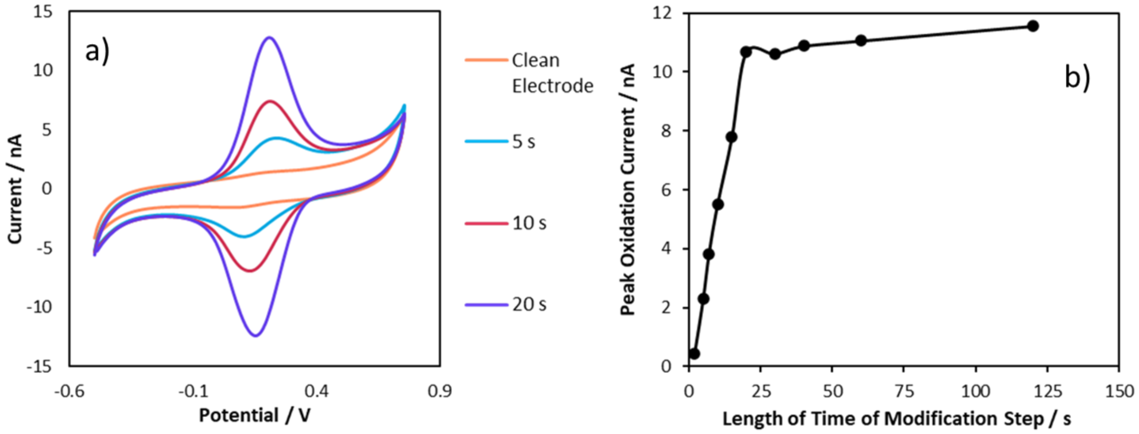

Figure S7. (a) Cyclic Voltammograms in 0.1 M NaCl (0.1 V s^{-1}) for pyridine-functionalised GC electrodes modified by constant potential at -2 V in 10 mM trifluoroacetylpyridinium salt for different lengths of time and then immersed in 1 mM ferrocyanide solution for 5 min: modification time 0 s (orange); 5 s (blue); 10 s (red) and 20 s (purple); (b) peak oxidation current for CV recorded in 0.1 M NaCl (0.1 V s^{-1}) for electrodes after modification at -2 V in trifluoroacetylpyridinium salt for different lengths of time, followed by immersion in 1 mM ferrocyanide for 5 min.

GC electrodes were modified by constant potential at -2 V vs. Fc/Fc^+ in 10 mM trifluoroacetyl-pyridinium in 0.1 M TBAPF6 for different lengths of time (see figure legend). The modified electrode was rinsed in acetonitrile, dried and then immersed in 1 mM ferrocyanide in 0.1 M NaCl for 5 mins to allow surface adsorption of the negatively charged redox probe to take place. The electrode was then transferred to 0.1 M NaCl solution and CV recorded at 0.1 V s-1, as shown in Fig S7. The unmodified electrode shows a featureless response, as expected for a 0.1 M NaCl solution that does not contain a redox active species. After modification the electrode exhibits a fairly symmetric redox response that is very characteristic of a surface confined species that can undergo reversible electron transfer; thus it is clear that ferrocyanide has been adsorbed by the pyridine film. Peak height of the CV increases with modification time for times 20 s or shorter, indicating that film thickness (and hence ability to take up the ferrocyanide) increases with the time that the electrode is modified for. However beyond 20 s modification time there is no further increase in peak height, indicating that the maximum film thickness is reached after 20 s. This is consistent with the limit in film formation noted when CV is used as the modification method.

10. Response of pyridine-modified boron doped diamond (BDD) to different redox probes

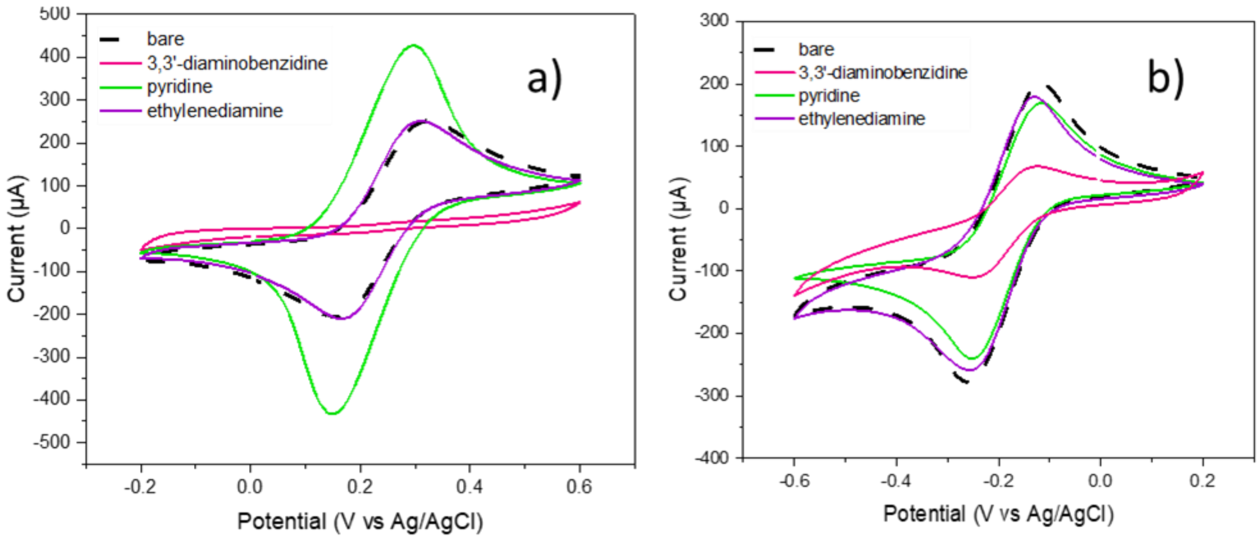

Figure S8: CVs at 0.1 V s^{-1} in a) 1mM ferrocyanide in 0.1 M pH 7 PBS at BDD working electrode unmodified (black dashed) and modified with trifluoroacetylpyridinium film as described in the main text (green); b) 1mM hexaammineruthenium in 0.1 M pH 7 PBS at BDD working electrode unmodified (black dashed) and modified with trifluoroacetylpyridinium film as described in the main text (green). Other modifications shown in pink and purple are not relevant to this study.

Similar to GC when modified with the pyridine/pyridinium layer, an enhancement in current is observed for the functionalised BDD in the negatively charged redox probe. This indicates that the surface is similarly modified. However the current enhancement is not as dramatic as seen for modified GC, which shows that the film is likely not so thick on the BDD compared to GC. This is consistent with Fig S2 c, where the passivation of the CV response in TFAA + pyridine after 9 scans was not complete for BDD, in comparison to GC where complete passivation was observed. This is supported by the CV response in 1 mM hexaammineruthenium (Fig S8 b), where the current for this positively charged redox probe is decreased for the functionalised BDD compared to the clean electrode, but the suppression of the response is very limited compared to observed for GC (see main manuscript). Thus we conclude that the film is not so dense of thick at BDD compared to other carbons, which is consistent with the lower reactivity of the diamond surface, making it less susceptible to grafting and modification by the pyridinium radical species.

11. Example of use of Henderson-Hasselbalch equation to determine [pyrH+] as a function of pH

The concentration of protonated pyridinium species [pyrH+] present at different pH values from the equilibrium:

$$\text{pyr} + \text{H}^+ \leftrightarrow \text{pyrH+}$$

can be estimated using the Henderson-Hasselbalch equation:

$$\text{pH}=\text{pK}_\text{a}+\log_{10}\left(\frac{[\text{pyr}]}{[\text{pyrH+}]}\right)$$

This can be rearranged:

$$[\text{pyrH+}] = \frac{[\text{pyr}]}{10^{\text{pH - pK}_\text{a}}}$$

As [pyr] is unknown, to obtain a normalised quantity of [pyrH+] we assume that:

$$[\text{pyr}]+[\text{pyrH+}]=1$$

Values for pKa of different substituted pyridine molecules were taken from literature as discussed in the main text.

12: XPS of Modified Electrode Treated with NaOH

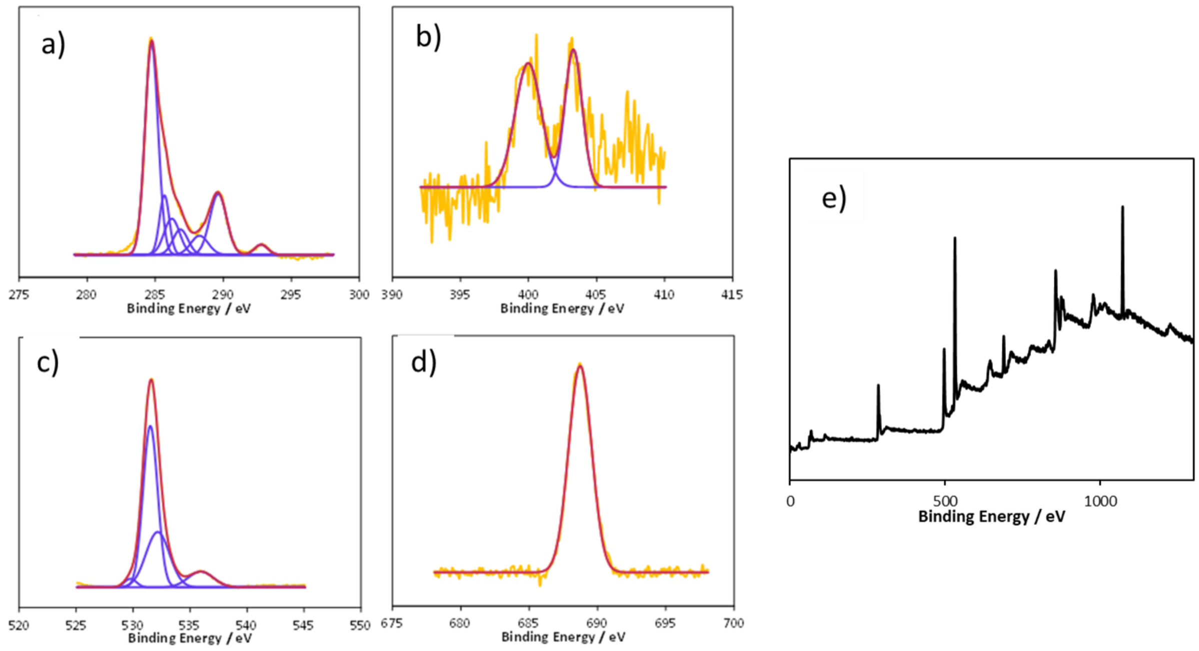

Figure S9: XPS data of a carbon electrode modified from trifluoroacetylpyridinium, then base-treated, showing the a) C1s region; b) N1s region; c) O1s region; d) F1s region; (Red line – cumulative fit; blue line – individual peaks; yellow line – raw data); e) survey spectrum.

13. XPS of Modified Electrode Treated with H_2SO_4

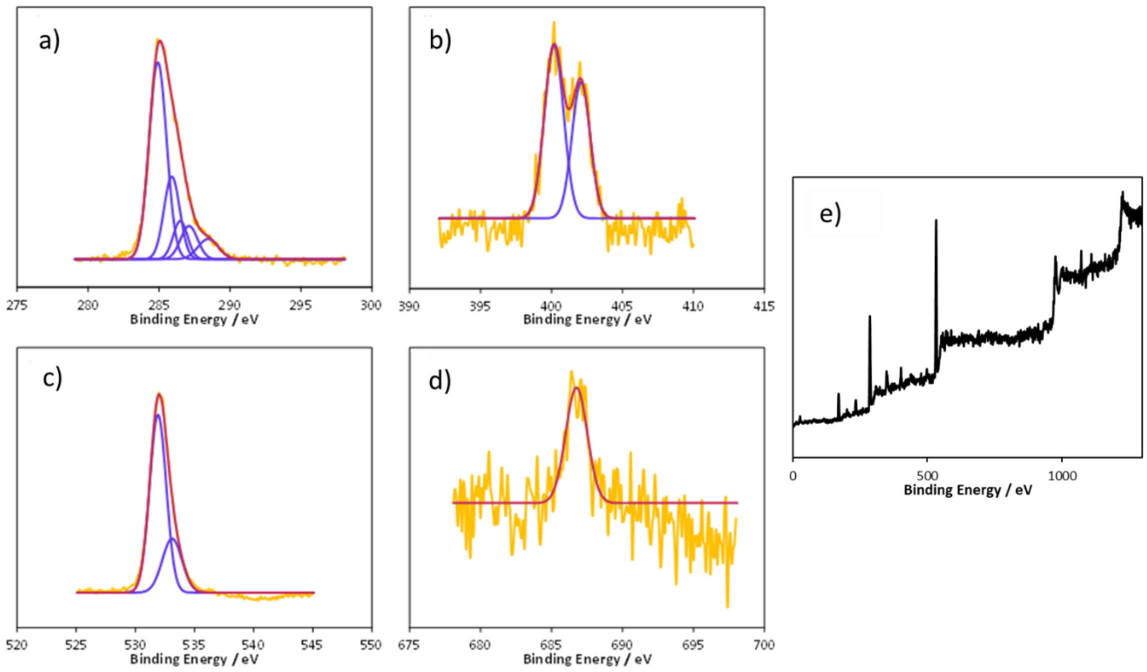

Figure S10: XPS data of a carbon electrode modified from trifluoroacetylpyridinium, base-treated then acid-treated showing the a) C1s region; b) N1s region; c) O1s region; d) F1s region; (Red line – cumulative fit; blue line – individual peaks; yellow line – raw data); e) survey spectrum.

14. CO_2 reduction using pyridine modified carbon electrodes – preliminary solution product analysis

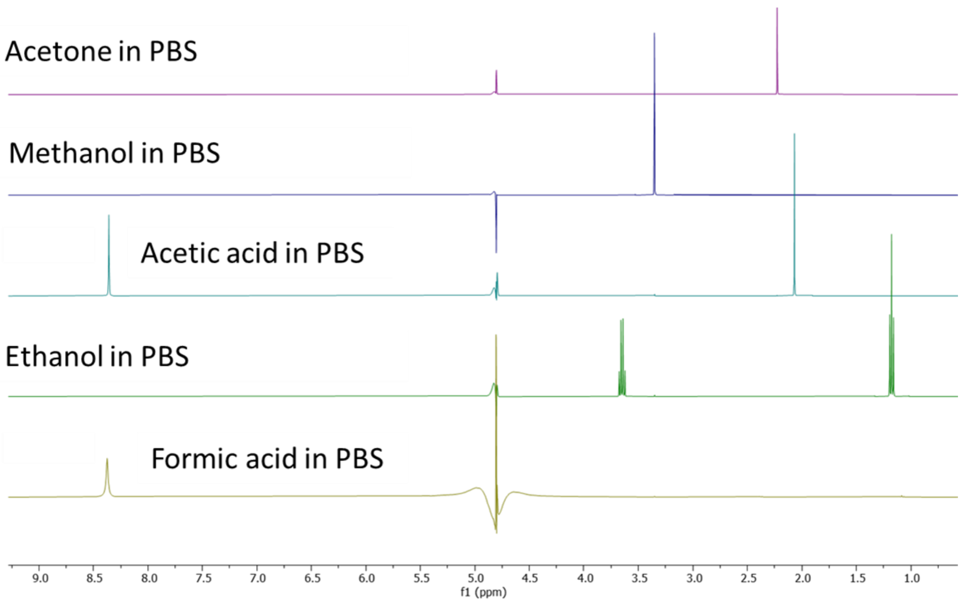

Figure S11: Proton NMR spectra for different solution standards (PBS containing different possible solution products from CO_2 reduction) to aid in product analysis.

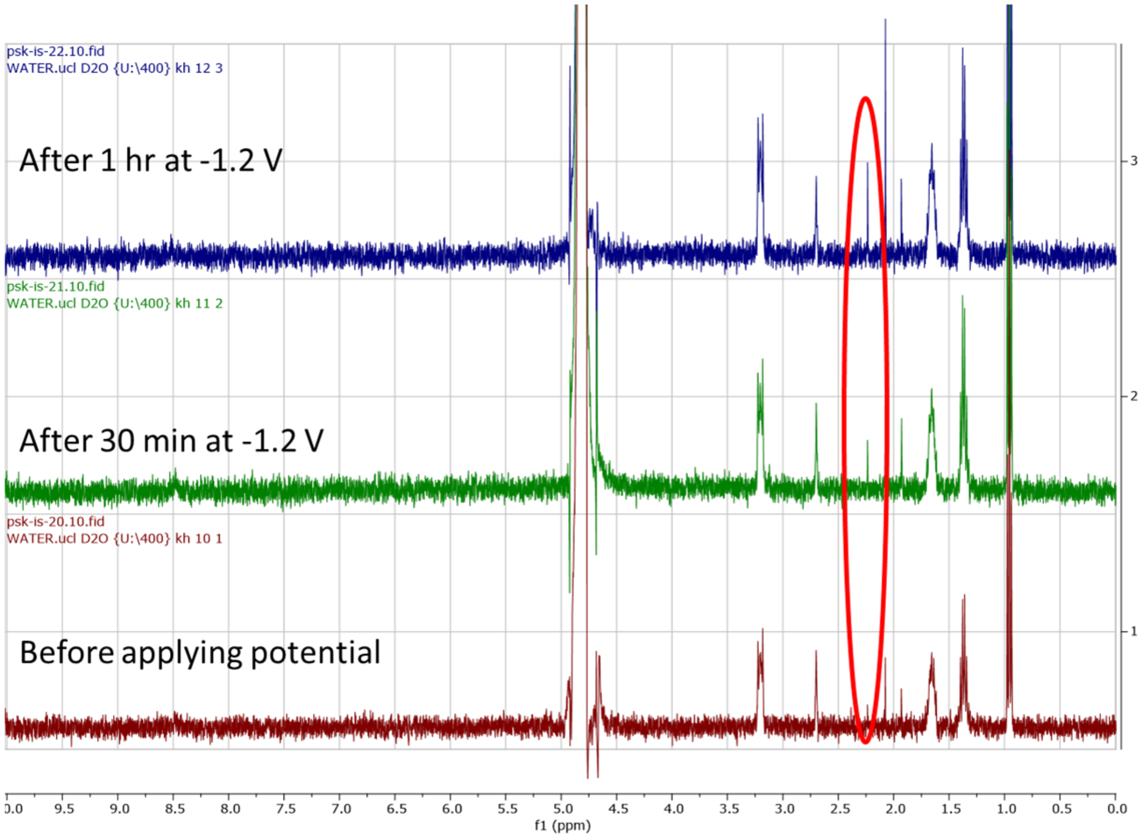

Figure S12: Proton NMR spectra of CO_2 saturated PBS solutions before and after 30 mins and 1 hrs of application of -1.2 V with pyridine / pyridinium modified graphite electrode. Signal corresponding to acetone at ca. 2.3 ppm is circled in red; note that peak for ethanoic acid at ca. 2.1 ppm also increases.

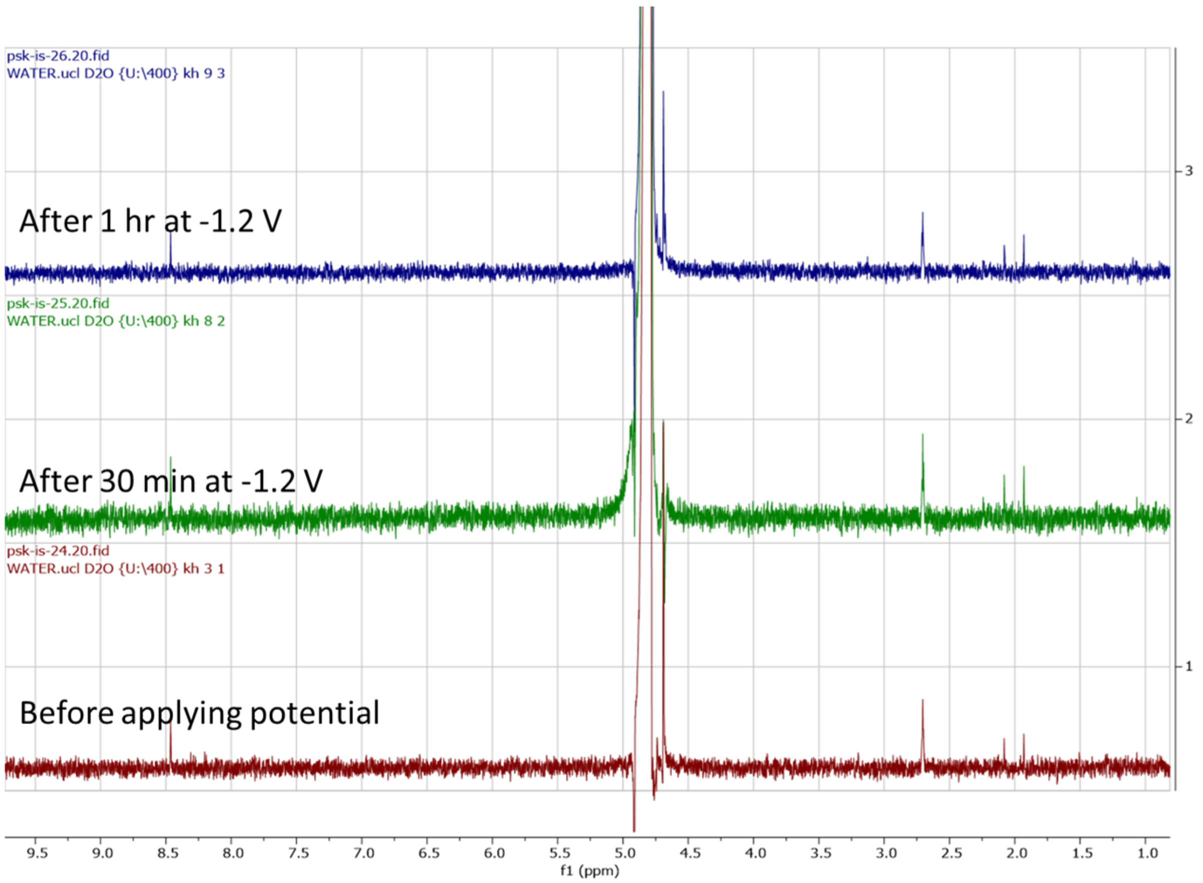

Figure S13: Proton NMR spectra of CO_2 saturated PBS solutions before and after 30 mins and 1 hrs of application of -1.2 V with unmodified graphite electrode. Signal corresponding to acetone at ca. 2.3 ppm is not present; small peaks close to background at ca. 2.7, 2.1 and 1.9 ppm suggests solution contamination which does not change with applied potential.

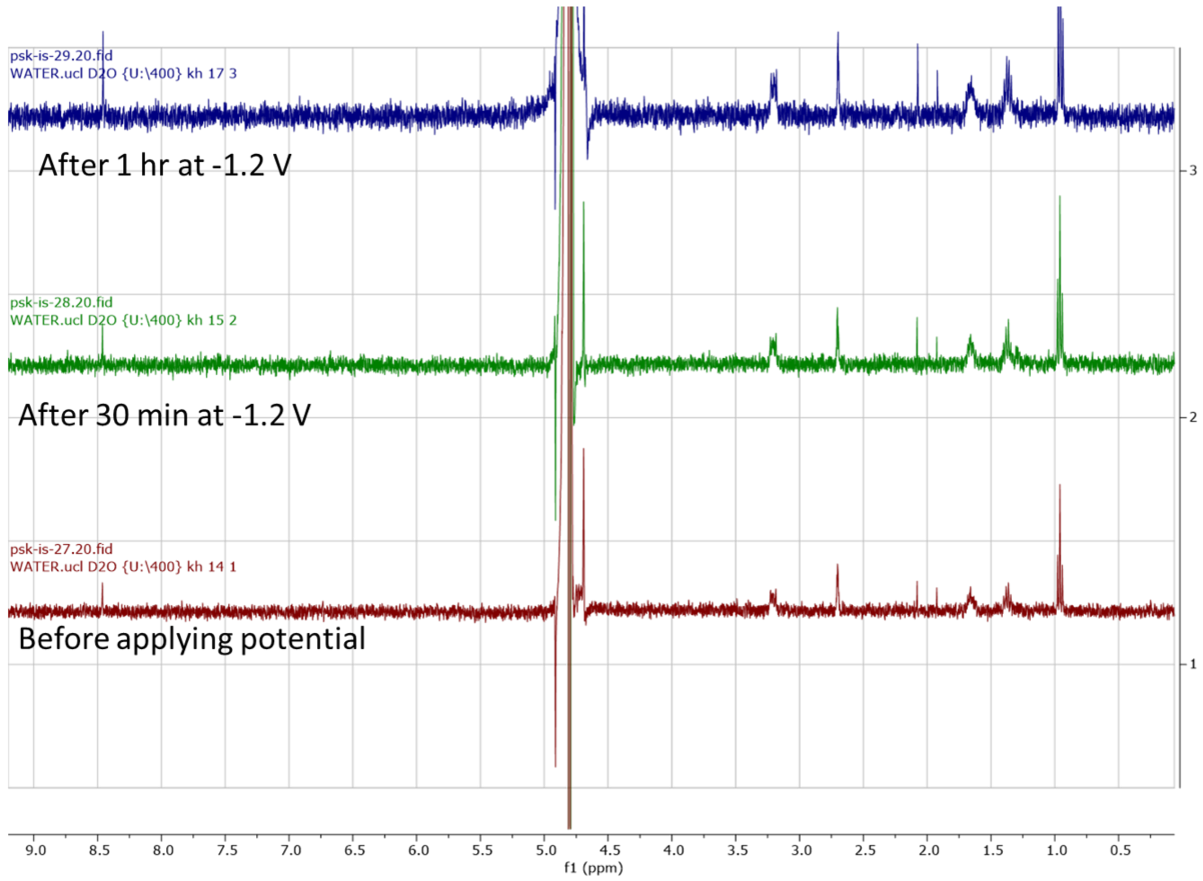

Figure S14: Proton NMR spectra of argon saturated PBS solutions before and after 30 mins and 1 hrs of application of -1.2 V with pyridine / pyridinium modified graphite electrode. Signal corresponding to acetone at ca. 2.3 ppm is not present; other peaks correspond to potential reduction products from film itself but are different from those seen in CO_2 saturated solution.
